# Molecular and genomic characterisation of a panel of human anal cancer cell lines

**DOI:** 10.1038/s41419-021-04141-5

**Published:** 2021-10-18

**Authors:** Glen R. Guerra, Joseph C. Kong, Rosemary M. Millen, Matthew Read, David S. Liu, Sara Roth, Shienny Sampurno, Joseph Sia, Maria-Pia Bernardi, Timothy J. Chittleborough, Corina C. Behrenbruch, Jiasian Teh, Huiling Xu, Nicole M. Haynes, Jiaan Yu, Richard Lupat, David Hawkes, Natasha Di Costanzo, Richard W. Tothill, Catherine Mitchell, Samuel Y. Ngan, Alexander G. Heriot, Robert G. Ramsay, Wayne A. Phillips

**Affiliations:** 1 Division of Cancer Research, Peter MacCallum Cancer Centre, Melbourne, VIC 3000 Australia; 2 Division of Cancer Surgery, Peter MacCallum Cancer Centre, Melbourne, VIC 3000 Australia; 3 The Sir Peter MacCallum Department of Oncology, The University of Melbourne, Parkville, VIC 3010 Australia; 4 Department of Clinical Pathology, The University of Melbourne, Parkville, VIC 3010 Australia; 5 Department of Surgery, St Vincent’s Hospital, The University of Melbourne, Parkville, VIC 3010 Australia; 6 UGI Surgery Unit, Austin Hospital, 145 Studley Road, Heidelberg, Victoria 3084 Australia; 7 Division of Radiation Oncology, Peter MacCallum Cancer Centre, Melbourne, VIC 3000 Australia; 8 Department of Pathology, Peter MacCallum Cancer Centre, Melbourne, VIC 3000 Australia; 9 Department of Biochemistry and Pharmacology, The University of Melbourne, Parkville, VIC 3010 Australia; 10 VCS Foundation, Carlton, VIC 3053 Australia; 11 Department of Pathology, University of Malaya, Kuala Lumpur, Malaysia; 12 Centre for Cancer Research, The University of Melbourne, Parkville, VIC 3010 Australia

**Keywords:** Targeted therapies, Anal cancer, Immune evasion, Cancer models

## Abstract

Anal cancer is a rare disease that has doubled in incidence over the last four decades. Current treatment and survival of patients with this disease has not changed substantially over this period of time, due, in part, to a paucity of preclinical models to assess new therapeutic options. To address this hiatus, we set-out to establish, validate and characterise a panel of human anal squamous cell carcinoma (ASCC) cell lines by employing an explant technique using fresh human ASCC tumour tissue. The panel of five human ASCC cell lines were validated to confirm their origin, squamous features and tumourigenicity, followed by molecular and genomic (whole-exome sequencing) characterisation. This panel recapitulates the genetic and molecular characteristics previously described in ASCC including phosphoinositide-3-kinase (PI3K) mutations in three of the human papillomavirus (HPV) positive lines and *TP53* mutations in the HPV negative line. The cell lines demonstrate the ability to form tumouroids and retain their tumourigenic potential upon xenotransplantation, with varied inducible expression of major histocompatibility complex class I (MHC class I) and Programmed cell death ligand 1 (PD-L1). We observed differential responses to standard chemotherapy, radiotherapy and a PI3K specific molecular targeted agent in vitro, which correlated with the clinical response of the patient tumours from which they were derived. We anticipate this novel panel of human ASCC cell lines will form a valuable resource for future studies into the biology and therapeutics of this rare disease.

## Introduction

Anal cancer is a rare disease that has more than doubled in incidence over the last four decades with limited improvements in treatment [1–4]. Consequently, the 5-year overall survival for patients with ASCC has remained almost static at 69% [2]. Squamous cell carcinoma accounts for ~85% of all histological types of anal cancer, with greater than 90% being HPV positive, forming a genetically distinct group of tumours with a better prognosis than viral negative ASCC [5–7]. Definitive chemoradiotherapy has remained the standard of care for non-metastatic ASCC since its inception four decades ago, with surgery relegated to a salvage role for those patients with locally persistent or recurrent disease [8–10]. For those with unresectable or metastatic disease, very limited palliative treatment options exist [11].

Due to the rarity of this disease, recruitment for clinical trials to investigate new therapeutic options remains a challenge [11]. Consequently, robust preclinical investigation is key to further our understanding of the pathogenesis of ASCC and identify which therapeutic options strongly merit further investigation in clinical trials [12]. Currently, there are limited models available to investigate the underlying biology of ASCC or for the preclinical testing of new therapies [12, 13].

We have established a panel of five human ASCC cell lines that cover the spectrum of both disease stage and treatment response. Here we present the validation and characterisation of these cell lines demonstrating that the panel recapitulates the published genomic and functional characteristics of ASCC.

## Results

### Establishment of anal cancer cell lines

We used a tumour explant approach to establish human ASCC cell lines. Of the fifteen patients that were recruited for the study, only eight patients had either adequate primary tumour tissue or successful xenograft implantation (to expand the primary tissue) for an attempt at deriving a cell line (Supplementary Table 1). Fresh tumour tissue (direct or patient-derived xenograft (PDX)) from the eight ASCC patients was explanted and maintained in culture until the epithelial component (Supplementary Fig. 1A) could be reliably passaged. Using this approach, long-lived (>50 passages) epithelial cell lines that could be cryopreserved and successfully recovered, were established from five ASCC patient samples. The lines were named sequentially as PMAC1–5 (Peter MacCallum Anal Cancer 1–5). Clinical data for the patients from which the cell lines were established can be found in Table 1. Three cell lines (PMAC1, 2 and 3) were derived from patients with treatment naive primary cancers who went on to achieve a complete response to treatment, and two (PMAC4 and 5) from patients with relapsed disease, both of whom subsequently succumbed to their disease.

**Table 1:** Cell line origin: patient demographics, tumour characteristics, treatment and response.

Cell line	Age/sex risk factors	Location	HPV type	Differentiation	LVI/PNI	Stage	Origin	Treatment	Response/outcome
PMAC1	58 M MSM (HIV−) Ex-smoker	Canal	+/16	Moderate HSIL adjacent	Nil	T2N0 (IIA)	Primary	5FU/MMC IMRT—54 Gy/30#	Complete Alive 5 y
PMAC2	45 F Non-smoker	Canal	+/16	Moderate Frequent mitoses Focal keratinisation	Nil	T2N1 (IIIA)	Primary	5FU/MMC IMRT—54 Gy/30#	Complete Alive 7 y
PMAC3	46 F Ex-smoker	Canal	+/16	Poor Basaloid, SCCis	LVI	T3N1 (IIIC)	Primary	5FU/MMC IMRT—54 Gy/30#	Complete Alive 5 y
PMAC4	46 M Smoker	Canal	+/16	Moderate to poor Frequent mitoses Focal keratinisation	LVI/PNI	T3N1 (IIIC)	Local Relapse	5FU/MMC IMRT—54 Gy/30# Palliative chemo Carboplatin/Paclitaxel	Locoregional + distant failure Died 20 m
PMAC5	51 F Ex-smoker	Canal	–	Moderate to poor Focal keratinisation	LVI++/PNI	T1N0 (I)	Local Relapse	Incidental excision Capecitabine/MMC VMAT—54 Gy/30# Salvage APR	Locoregional failure Died 2 y

*PMAC* Peter MacCallum Anal Cancer, *M* male, *F* female, *MSM* men having sex with men, *HSIL* high grade squamous intraepithelial lesion, *5FU* 5-fluorouracil, *MMC* mitomycin C, *IMRT* intensity modulated radiotherapy, *VMAT* volumetric modulated arc therapy, *#* fractions, *SCCis* squamous cell carcinoma in situ surrounding invasive.

In all five cell lines, short tandem repeat analysis confirmed the relationship between the cell line and the matched parent tumour with 89–100% conservation across 10 loci (Supplementary Table 2). All lines were confirmed to be free of mycoplasma by PCR (Supplementary Fig. 1B).

**Fig. 1:**
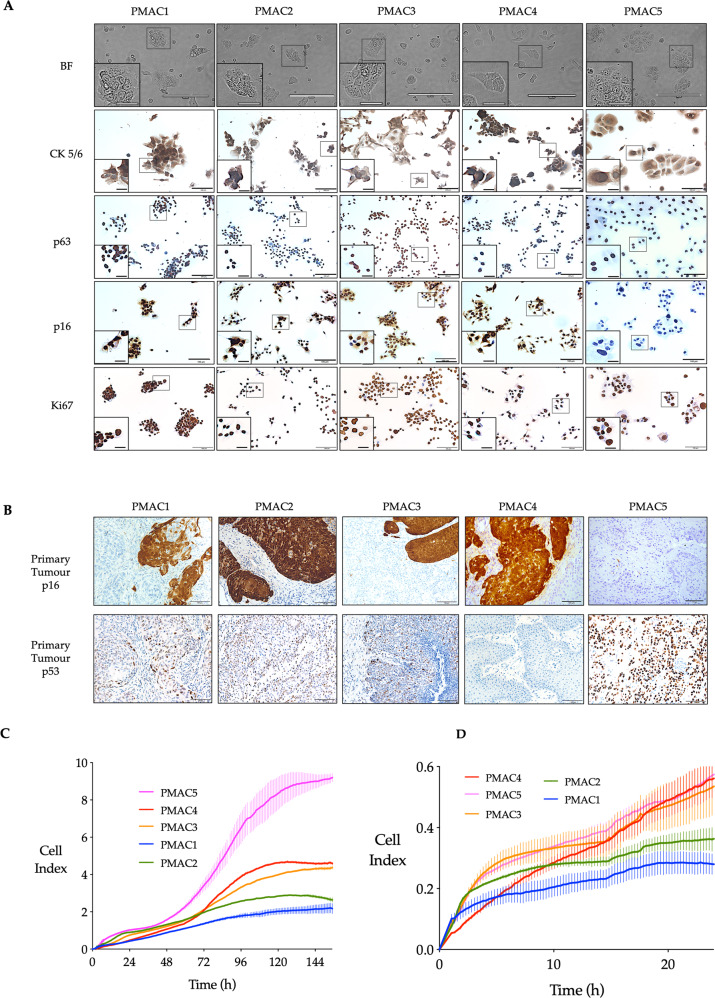
Characterisation of anal SCC cell lines in vitro. **A** Brightfield photomicrographs (scale bar 200 μm; inset 50 μm) of the five human ASCC cell lines 48 h following seeding. Cells were then fixed with paraformaldehyde and permeabilised with Triton X before immunocytochemical staining for CK5/6, p63, p16 & Ki67 (scale bar 100 μm, inset 25 μm). **B** Immunohistochemical assessment of p16 and p53 expression in the parent human tumours from which the cell lines were derived (scale bar 100 μm). **C** Proliferation assay demonstrating the real time cell index recorded over a period of 150 h for each cell line seeded (PMAC1–3 (2 × 10^4^ cells), PMAC4 and 5 (1 × 10^4^ cells)) in RPMI 1640 + 10% FCS in an xCELLigence DTPA 16 well E-plate (Mean ± SEM depicted across each time point, *n* = 2, separate independent experiments in triplicate). **D** Migration assay towards 20% FCS from serum starved media, demonstrating the real time cell index recorded over a period of 24 h for each cell line seeded (PMAC1–3 (8 × 10^5^ cells) PMAC4 and 5 (4 × 10^5^ cells)) in an xCELLigence DTPA 16 well E-plate (Mean ± SEM depicted for each time point, *n* = 2, separate independent experiments in triplicate).

All cell lines grew as an adherent monolayer in vitro and demonstrated a classic pavement appearance with subtle differences in cell and colony size and morphology between the lines on brightfield microscopy (Fig. 1A). All lines demonstrated appropriate expression of cytokeratin 5/6 (CK 5/6) and p63 on immunocytochemical (ICC) staining, consistent with cells of squamous origin (Fig. 1A) [14]. The marker of cell proliferation, Ki67, was expressed in >90% of cells across all lines on ICC, consistent with an exponential phase of growth (Fig. 1A) [15].

Assessment of p16 expression with ICC demonstrated strong nuclear and cytoplasmic staining in all lines except PMAC5 (Fig. 1A), consistent with the p16 expression in the parental tumours on immunohistochemistry (IHC) (Fig. 1B). HPV genotyping of the cell lines, demonstrated the four p16 expressing lines harbour HPV 16 alone out of up to 37 genotypes assessed across six commercial polymerase chain reaction (PCR) based assays. The PMAC5 line was HPV negative across all six assays, consistent with its p16 negative status [16]. Aberrations in *TP53* are a frequent finding in HPV negative ASCC [5]. Parental tumour expression of p53 on IHC was normal in three of the p16 positive tumours, absent in the remaining p16 positive tumour (PMAC4), and increased in the HPV negative tumour (PMAC5) (Fig. 1B). The lack of p53 staining in the tumour from which PMAC4 derived was confirmed by including positive and negative controls to exclude technical failure. Furthermore, mild staining consistent with normal *TP53* expression was detected in the stromal component of the specimen.

### Growth characteristics

Cell culture characteristics differed significantly between the lines, with those developed from the three patients achieving a complete response having a lower seeding efficiency than PMAC4 and 5 (Fig. 1B). Proliferation and migration assays were performed over 150 and 24 h, respectively (Fig. 1C, D). The two lines established from patients with relapsed disease (PMAC4, PMAC5) demonstrated the most rapid doubling time and greatest migration potential (Supplementary Table 3).

**Fig. 2:**
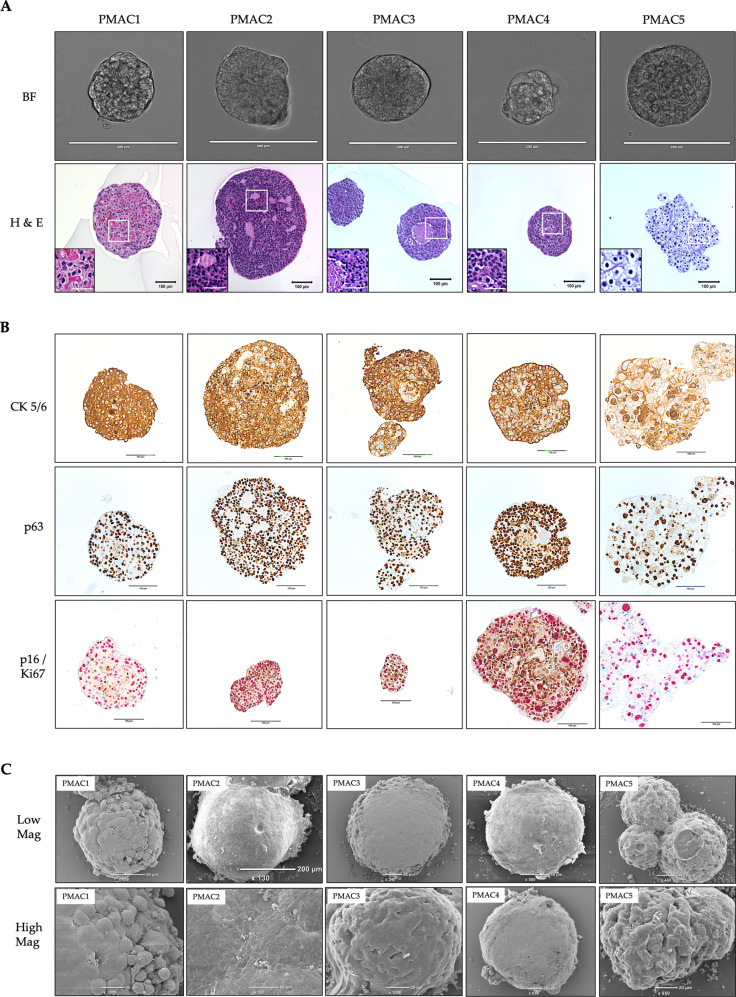
Characterisation of anal SCC cell lines as 3D tumouroids. **A** Brightfield photomicrographs (scale bar 200 μm) and haematoxylin and eosin staining (scale bar 100 μm; inset 50 μm) of cell line tumouroids 14 days following seeding of cells in Matrigel^TM^. **B** Tumouroids were fixed with paraformaldehyde, embedded in histogel, sectioned and stained for CK5/6, p63 & p16 (brown chromagen)/Ki67 (red chromagen). **C** Surface architecture of cell line tumouroids assessed following growth for 14 days, retrieval, fixation and processing for scanning electron microscopy (ScEM). (top row: low magnification, scale bar 50 μm except PMAC2 200 μm; bottom row: high magnification, scale bar 20 μm, except PMAC2 50 μm).

**Fig. 3:**
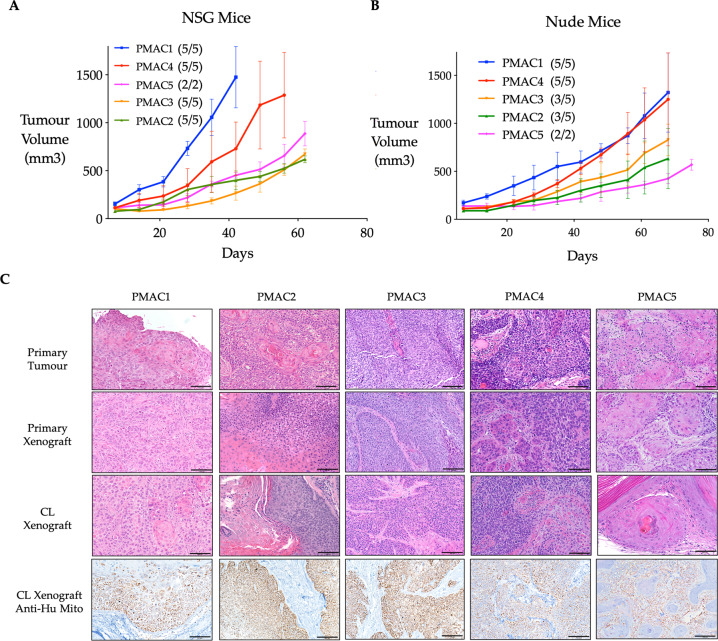
Characterisation of anal SCC cell lines as xenografts. Tumourigenicity of the five human ASCC lines was assessed in both **A** NSG and **B** athymic (Nude) mice following subcutaneous injections of 5 × 10^6^ cells in 100 μl of 1:1 Matrigel:PBS. The tumours were measured with the growth demonstrated (Mean ± SEM) of those mice with tumours for each cell line. The proportion of mice developing established tumours is displayed in the key. **C** Histological architecture of the five human ASCC tumours and the derived tumour models following formalin fixation, paraffin embedding, sectioning and staining (Scale bar 100 μm). The haematoxylin and eosin images demonstrate preservation of the cellular and architectural features of the human ASCC tumours from which the primary and cell line xenografts were subsequently derived. Anti-human mitochondrial immunohistochemistry demonstrates staining of the human epithelial tumour cells, with absent staining of the mouse stroma, validating the mouse cell line tumours as human in origin. CL, cell line; Anti-Hu Mito, anti-human mitochondrial.

In addition to 2D growth, all the cell lines were also capable of forming 3D tumouroids when grown in Matrigel. Under bright field microscopy, the cell line-derived tumouroids demonstrated similar size and a non-budding spheroid morphology (Fig. 2A). On haematoxylin and eosin staining, they retained the histologic characteristics of squamous carcinomas and the parent tumour, with differences in the cellular morphology, micro-architecture and degree of extracellular keratin between the lines (Figs. 2A, 3C). The IHC staining profile for CK5/6, p63, p16 and Ki67 was consistent with the cell line expression and that of squamous cell carcinomas (Fig. 2B). Scanning electron microscopy revealed the tumouroids to have a spherical shape composed of tightly spaced cells, with varying morphology evident between the lines (Fig. 2C).

### Tumourigenic potential

To assess the tumourigenic potential of the lines, cells were subcutaneously injected into immunocompromised mice. After 6 weeks, this yielded macroscopically visible tumours in all NSG (most immunodeficient), and most BALB/c Nude (least immunodeficient), mice (Fig. 3A, B). Haematoxylin and eosin staining of the tumours revealed variable keratinisation and the tumour cells displayed features consistent with a carcinoma (Fig. 3C). Comparison of the matched cell line xenografts, original tumour and PDXs revealed the maintenance of tumour-specific histopathological changes (Fig. 3C). Staining for anti-human mitochondrial antigen, confirmed the cell line xenografts as human in origin (Fig. 3C).

### Sequencing analyses

**Fig. 4:**
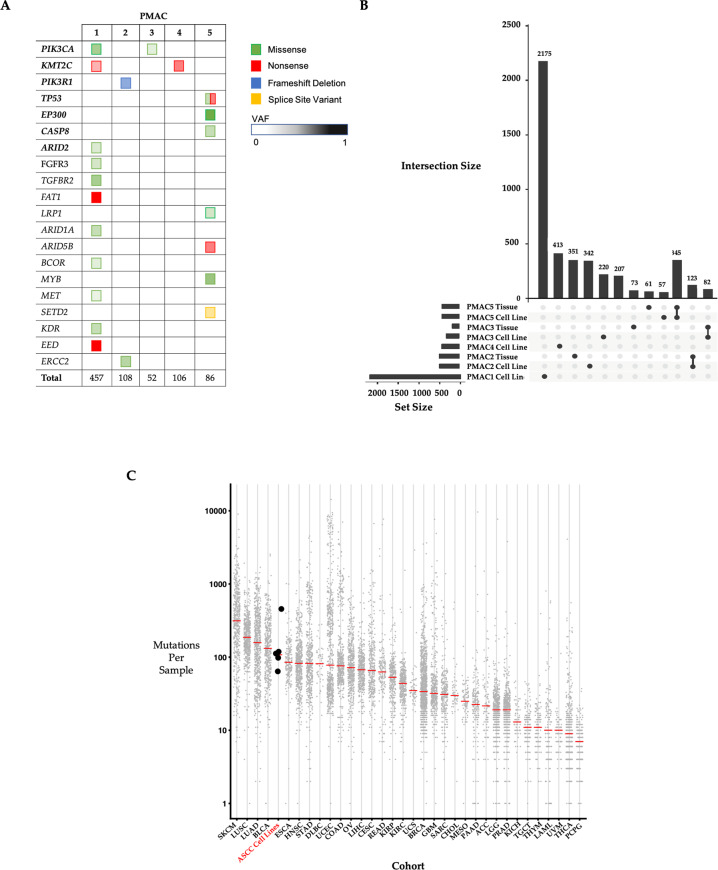
Genomic characterisation of somatic variants and mutational load in the anal SCC cell lines and parent tumours. **A** Summary of the 20 most frequent and relevant somatic mutations in each of the five ASCC cell lines generated from whole-exome sequencing (WES). The colour of the square indicates the type of mutation detected, with the intensity of the colour indicating the variant allele frequency (VAF) of the detected genomic aberration. Those listed in bold are amongst the twenty most frequently observed mutations in ASCC, with the remaining genes all having been previously reported to harbour mutations in ASCC. Empty boxes indicate absence of the variant. Total indicates the total number of non-synonymous somatic variants detected in each cell line. **B** Plot of unique and shared somatic mutations (synonymous and non-synonymous) across the panel of ASCC cell lines and parent tumour samples (Set size = total number mutations, Intersection size = shared mutations between samples identified by the linked dots or unique to the sample alone (single dot)). **C** The mutational burden of the panel of ASCC cell lines is plotted against the mutational burden of the other TCGA cohorts (https://www.cancer.gov/tcga), with the median of the ASCC panel sitting at a similar level to head and neck SCC (HNSC). (black dots, ASCC cell lines; grey dots, individual patients; Red bar, median; SKCM, melanoma; LUSC, lung SCC; LUAD, lung adenocarcinoma; BLCA, bladder cancer; ESCA, esophageal SCC; HNSCC, head and neck SCC; STAD, stomach adenocarcinoma; DLBC, diffuse large B cell lymphoma; UCEC, uterine corpus endometrial carcinoma; COAD, colorectal adenocarcinoma; OV, ovarian cancer; LIHC, liver hepatocellular carcinoma; CESC, cervical SCC; READ, rectal adenocarcinoma; KIRP, kidney renal papillary cell carcinoma; KIRC, kidney renal clear cell carcinoma; UCS, uterine carcinosarcoma; BRCA, breast carcinoma; GBM, glioblastoma multiforme; SARC, sarcoma; CHOL, cholangiocarcinoma; MESO, mesothelioma; PAAD, pancreatic adenocarcinoma; ACC, adrenocortical carcinoma; LGG, low grade glioma; PRAD, prostate adenocarcinoma; KICH, kidney chromophobe; TGCT, testicular germ cell tumours; THYM, thymoma; LAML, acute myeloid leukaemia; UVM, uveal melanoma; THCA, thyroid carcinoma; PCPG, phaeochromocytoma and paraganglioma).

Deoxyribonucleic acid (DNA) from each of the cell lines underwent whole-exome sequencing, with somatic variants identified by comparing to matched peripheral blood mononuclear cell (PBMC) DNA. Across the panel of cancer cell lines, seven of the twenty most common genomic aberrations in ASCC [17–20] were identified, with each line harbouring at least one of these variants (Fig. 4A). Mutations in phosphoinositide 3-kinase genes were detected in three of the cell lines; missense activating *PIK3CA* (NM_006218.2) mutations *E726K* and *Q546P* in PMAC1 and PMAC*3*, respectively, and a frameshift inactivating mutation E458* in *PIK3R1* (NM_170606.2) in PMAC2. Two cell lines harboured nonsense *KMT2C* (NM_170606.2) mutations, Q88* (PMAC1) and E1750* (PMAC4). Mutations were also identified in both alleles of *TP53* (NM_000546.5) in PMAC5, one a truncating nonsense mutation (p.R213*) and the other a gain-of-function missense mutation (*G279W*). Identical mutations were identified in the parent tumours where such sequencing was performed (PMAC2, PMAC3 and PMAC5). In spite of the total lack of p53 protein expression in PMAC4 (Fig. 1B), no mutation in *TP53* or its splice sites was detected in this cell line, or its parental tumour (using a targeted panel which included all exons and splice sites of the *TP53* gene). The lack of p53 protein expression in the absence of a genomic mutation is not uncommon [21] and may potentially be explained by epigenetics mechanisms such as *TP53* promoter methylation.

A comparison of the somatic mutations between PMAC2, PMAC3 and PMAC5 and their respective parental tumours revealed significant overlap (Fig. 4B). PMAC1 and PMAC4 could not be compared with their parental tumours due to insufficient tumour remaining for sequencing. There was minimal overlap between the non-matched samples. There was also a similar pattern of single nucleotide variants and small insertions and deletions between the cell lines and their respective parent tumours (Supplementary Fig. 2).

Assessment of mutational burden revealed four cell lines (mutations/Mb = PMAC5 4.39, PMAC2 4.26, PMAC4 4.13, PMAC3 2.88) with a similar somatic burden to the mean of 5.7 mutations/Mb published previously for a cohort of ASCCs [18]. The exception was the PMAC1 cell line, which had a much higher mutational load (19.86 mutations/Mb), but still within the reported range (0.8–35.5 mutations/Mb) for ASCC [18, 22]. The mutational burden was compared to other cancers from The Cancer Genome Atlas (TCGA) (Fig. 4C) [22], revealing the panel to possess a similar mutational load to head and neck SCC, which is also HPV associated and has a similar genomic landscape to ASCC [23].

### Copy number analysis

**Fig. 5:**
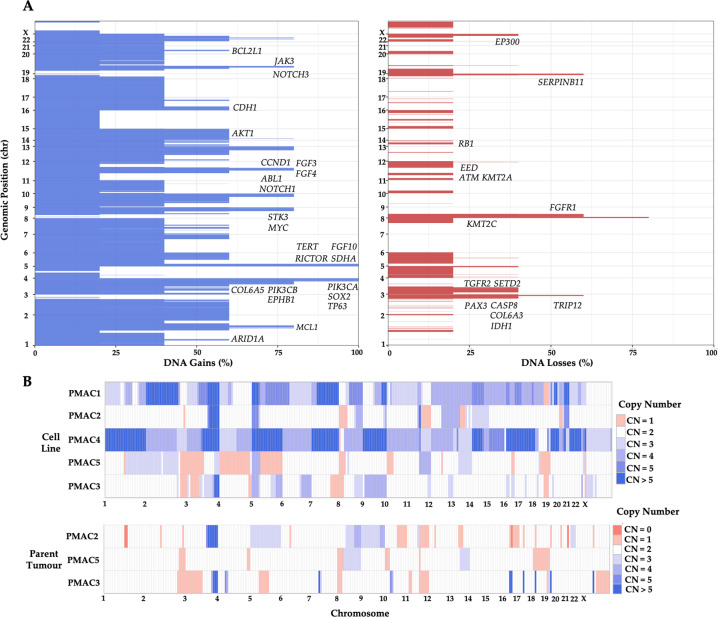
Genomic characterisation of copy number variants in the ASCC cell lines and parent tumours. **A** From the WES analysis, the percentage of the five ASCC cell lines with a copy number gain (red) or loss (blue) at each chromosomal region are presented. A gain or loss was defined by a mean log ratio above 0.3 or below −0.3, respectively when compared to the patient matched PBMC DNA. **B** The cytoband represents the copy number gains and losses across the chromosomal regions for each of the five ASCC cell lines and their respective parent tumours for PMAC2, 3 and 5 (CN = copy number).

Somatic copy number analysis (SCNA) revealed gains in 3q and 5p across all the lines (Fig. 5A), and this has been reported as a frequent event in ASCC [17]. These regions include the genes *PIK3CA*, *PIK3CB*, *TP63*, *SOX2*, *FGF10*, *TERT*, *RICTOR*, and *SDHA*. Other focal gains included regions harbouring genes with mutations reported in ASCC including *STK3*, *MYC*, *ABL1*, *NOTCH1*, *CCND1* and *BCL2L1* [17–19, 24].

The SCNA also identified shared significant losses in 2q and 8p and multiple focal losses across all five cell lines (Fig. 5A). These regions include the genes *TRIP12*, *COL6A3*, *IDH1*, *ERBB4*, *PAX3* and *FGFR1*. Other areas of focal loss included regions with mutations reported in ASCC including *FGFR1*, *MGMT*, *EED*, *ATM*, *NTRK3*, *SMAD4*, *ARID3A*, *SERPINB11* and *STK11*. All of these genes have been implicated as tumour suppressor genes with their loss suggesting a role in tumourigenesis. [17–19, 24]

The cytoband revealed gains to be more frequent than losses and no line losing both copies of a gene (Fig. 5B, Supplementary Fig. 3). PMAC1 and PMAC4 had multiple whole chromosomal gains and, in contrast to the other lines, PMAC5 was characterised by losses. There was significant consistency between the SCNA profiles in the cell lines and respective parental tumours for the three assessable lines (PMAC2, PMAC3 and PMAC5).

### Mutational trinucleotide signatures

Mutational trinucleotide signatures are characteristic combinations of mutation types arising from specific mutagenesis processes. Based on the classification by Alexandrov et al., all five cell lines were found to harbour more than one mutational trinucleotide signature (Supplementary Fig. 4) [25, 26]. Signature SBS1 was identified in four of the cell lines, related to an endogenous mutational process associated with age, and is consistent with a previous publication where 100% of assessed ASCC samples demonstrated the equivalent signature 1 (v2) [17, 25]. The activity of SBS1 is often correlated with signature SBS5, identified in two of the lines harbouring the SBS1 signature. Signature SBS2 was identified in three of the HPV positive lines and has been attributed to activity of the AID/APOBEC cytidine deaminases, with increased APOBEC mutagenesis in HPV positive cancers [27]. The APOBEC enzymes likely generate many of the driver mutations in HPV-associated cancers, being directly implicated in generating oncogenic helical domain *PIK3CA* mutations and, consequently, HPV-driven tumourigenesis [28]. Signature SBS13 has also been attributed to activity of the AID/APOBEC family and is commonly found with signature SBS2, as identified in PMAC1 and PMAC2 cell lines.

Signature SBS10b was identified in PMAC1 and 2 and has been linked with the generation of large numbers of mutations in a small subset of this signature, termed hypermutators [25]. This is consistent with the mutational load of PMAC1 being far above that of the other four cell lines, with PMAC2 having the second highest TMB (Fig. 4C). Pathogenic missense somatic mutations in *ARID1A* and *PALB2*, which are associated with a deficiency in homologous recombination [29], were identified in PMAC1 and may also partly explain its relatively high mutational load. Signature SBS7b which has been associated with ultraviolet light exposure, was identified in PMAC5 and has also been reported in other ASCC patient samples [17]. Signature SBS87 was identified in four, SBS24 in three, SBS6 in two and SBS15, SBS29 and SBS31 each in one cell line, none of which been reported in ASCC previously.

### Inducible expression of MHC class I and PD-L1

**Fig. 6:**
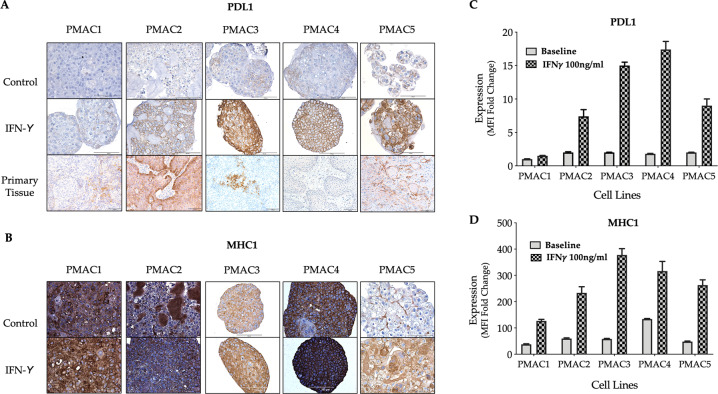
MHC I & PD-L1 expression in anal cell lines and cell line tumouroids. The panel of ASCC cell line tumouroids were grown in matrigel and RPMI medium containing 10% FCS. After 5 days in culture the tumouroids were exposed to IFN-γ (100 ng/ml) for 48 h, following which they were retrieved, fixed, embedded, sectioned and stained by IHC for **A** PD-L1 and **B** MHC I expression. Cells were counter stained with haematoxylin. The parent tumour tissue PD-L1 IHC staining was performed for comparison (Scale bar 100 μm). **C**, **D**. Expression of PD-L1 and MHC I was also assessed by flow cytometry. Following seeding of cells 24 h prior, expression was assessed after growth in the absence (plain bar) and presence (checked bar) of 100 ng/ml IFN-γ for 48 h. Results are expressed as the fold change of the mean fluorescent intensity (MFI) in relation to the respective isotype control (mean percentage of control ± SEM, *n* = 2).

Interferon-gamma (IFN-γ) is a key arbiter in inducing the anti-tumour effects of radiation, and given the role of MHC class I and PD-L1 in determining treatment response and outcomes for anal cancer patients [30–34], their expression was assessed by IHC and flow cytometry. At baseline, there was variable and patchy PD-L1 expression which was substantially upregulated on exposure to IFN-γ in all lines except PMAC1, which had minimal expression at baseline or with stimulation (Fig. 6A, B). PMAC4 was identified to have the highest inducible PD-L1 expression of the panel. Similar findings were identified in assessing the parent tumour specimens for PD-L1 with IHC, with the parent tumour of PMAC1, PMAC3 and PMAC 4 demonstrating stromal but not tumoural expression of PD-L1 (Fig. 6A). The parent tumour samples of PMAC 2 and PMAC 5 had moderate and high PD-L1 expression respectively, consistent with it being inducible as identified in vitro. There was no correlation between the mutational burden and PD-L1 expression across the panel, consistent with findings in other cancers (Supplementary Table 4) [35]. An assessment of CD3 tumour infiltrating lymphocytes by IHC in the parent tumour also failed to demonstrate a consistent correlation with the tumour mutational burden or PD-L1 expression.

From the quantitative flow cytometry data, baseline MHC class I expression was substantial across all cell lines. Following exposure to IFN-γ, there was upregulation of expression in all lines (Fig. 6C, D). IFN-γ was also found to have a direct effect on the viability and growth of the cell lines. The effect was most pronounced on the HPV negative line PMAC5, with a severe reduction in the size of the colonies and 35% reduction in the overall colony count. There was a moderate reduction in the size and count for PMAC2 and PMAC4 (72 and 64% of control colony count, respectively), with minimal change for PMAC1 and PMAC3 (colony count 92 and 95% of controls, respectively; data not shown).

### In vitro assessment of response to therapy

**Fig. 7:**
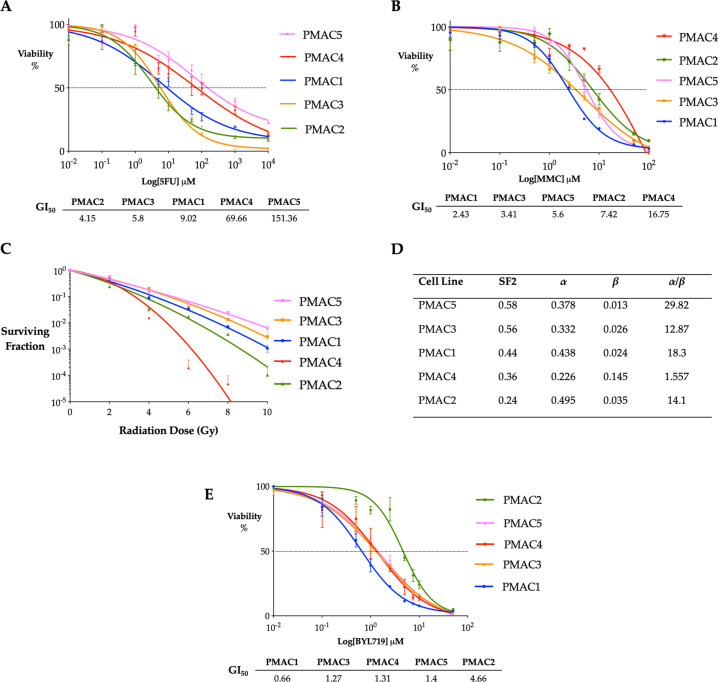
Chemotherapy, radiotherapy and molecular targeted therapy assessment in the ASCC cell lines. **A**, **B**. In vitro 5-FU and MMC cytotoxic assays. Dose–response curves for single agent treatment of the panel of ASCC cell lines for 96 h with viability quantified by an AlamarBlue^®^ assay. Data are expressed as percent of vehicle treated control. Shown are mean ± SEM from three independent experiments, GI_50_ presented below. **C** Radiation dose-response curves for the human ASCC cell lines. Radiation survival curves demonstrate the survival fraction (log10) following irradiation with 2, 4, 6, 8 and 10 Gy for the panel of five human ASCC cell lines (mean ± SEM from three independent experiments). **D** Data points were fitted with the linear quadratic equation, with the radiotherapy surviving fraction of 2 Gy (SF2) and the α and β values and α/β ratio for each cell line calculated. **E** Dose–response curve for treatment of the panel of ASCC cell lines with BYL719 for 96 h with viability quantified by an AlamarBlue^®^ assay. Data are expressed as percent of vehicle treated control. Shown are the mean ± SEM for three independent experiments, GI_50_ presented below.

To demonstrate the utility of the cell lines as a preclinical models for assessing potential therapies, we examined their sensitivity to two chemotherapeutic drugs employed in the primary treatment of patients with ASCC and compared the response of the cell lines to the clinical response observed in patients (Fig. 7A, B). This revealed that both relapsed lines (PMAC4 and PMAC5) had pronounced resistance to 5-fluorouracil (5-FU), while the lines established from patients who responded to primary treatment (PMAC1–3) demonstrated increased sensitivity. For Mitomycin C (MMC), PMAC4 demonstrated the greatest resistance, while PMAC2 and PMAC5 were the next most resistant followed by PMAC1 and PMAC3.

The sensitivity of the panel of ASCC lines to radiotherapy was assessed by determining the surviving fraction (SF) across 2–10 Gy to provide the SF of 2 Gy (SF2) and dose–response curves, derived from the linear quadratic equation [SF(D) = exp(−αD − βD^2^)] (Fig. 7C, D). The linear quadratic model describes the SF of clonogenic (or stem) cells as a function of single fraction radiation doses (D) [36]. The *α* and *β* parameters represent the intrinsic radiosensitivity of the cells with cell death resulting from the α (linear) component increasing linearly with the dose, compared with cell death from the β (quadratic) component, which increases in proportion to the square of the dose. Higher values for both indicate increased sensitivity to radiation. The ratio of both parameters *α/β* is a measure of the fractionation sensitivity, with a higher ratio indicating less sensitivity to the effect of fractionation. While there are several models to describe the radiobiological response, the linear quadratic has been best validated by both experimental and clinical data [36].

These data demonstrate the HPV negative line, PMAC5, to be the most resistant to radiotherapy in terms of SF2 and across increasing doses. This is consistent with the clinical perception that non-virally driven ASCC tumours are more radio-resistant, secondary to the high incidence of p53 mutations in this cohort [5, 37]. Indeed, whole-exome sequencing data confirmed PMAC5 harbours both a truncating and separate missense gain of function *TP53* mutation (Fig. 4A). Surprisingly, PMAC4, although derived from a treatment resistant tumour following chemoradiotherapy, was found to be radiosensitive on assessment of the SF2 parameter and also exquisitely sensitive to increasing single fraction doses, as reflected by the very low *α/β* ratio. The curves and *α/β* ratios had some similarity across the three complete response lines (PMAC1, PMAC2, PMAC3), with the SF2 lying along the range from sensitive to resistant, with PMAC3 notably having an SF2 of 0.56, close to that of PMAC5 (Fig. 7C, D).

Given the frequency of *PIK3CA* aberrations in ASCC [17–20], assessment of sensitivity across the panel of cell lines to the *PI3K*α-specific inhibitor BYL719 (Alpelisib) was performed (Fig. 7E). Four of the cell lines (PMAC1 & PMAC3, PMAC4 & PMAC5) have a GI_50_ in the sensitive to intermediate range when compared to head and neck SCC and *PIK3CA* mutant breast cancer cell lines [38, 39]. Examining those four lines, PMAC1 and PMAC3 harboured *PIK3CA* mutations and copy number gains at the *PIK3CA* locus, while PMAC4 and PMAC5 had copy number gains alone. PMAC2 demonstrated a 3-fold higher GI_50_ than the next highest line (PMAC5), falling into the resistant range based on published data [38].

## Discussion

Progress in understanding the biology of ASCC and the development of new therapeutic options has been severely hampered by the paucity of appropriate cell lines for in vitro and in vivo studies. In 2009, Takeda et al. reported the establishment and characterisation of a HPV negative ASCC cell line [40]. However, there have been no additional reports using this cell line and it does not appear to be available for further study. More recently, Wechsler et al. [41]. reported HPV-16 transformation of a human anal epithelial line that demonstrated some in vitro features suggestive of neoplasia, but it was not tumourigenic in mice (Nod-SCID-IL2Rγ^null^), and therefore not representative of established ASCC. To our knowledge, there are no other validated ASCC cell lines available.

Here we report the establishment, validation and characterisation of a panel of five novel human ASCC cell lines. As a new resource, they begin to address the current critical lack of preclinical models for this disease and will facilitate investigations into the underlying biology of ASCC and the evaluation of novel therapeutics.

These cell lines are representative of the spectrum of both disease stage and treatment response with, importantly, two lines established from treatment resistant relapsed tumours. One of these relapsed lines is derived from, and recapitulates, the HPV negative form of the disease, which is the most resistant form of ASCC and likely to become the predominant type encountered in future decades, once HPV vaccination has taken effect.

These cell lines grow in vitro as 2D monolayers, or 3D tumouroids when seeded in Matrigel^TM^, and all are tumourigenic in vivo, growing as cell line-derived tumour xenografts in two different immunocompromised mouse strains. They all also exhibit features consistent with ASCC, including morphology, cytokeratin expression and histological architecture, both in vitro and in vivo. Four of the lines (PMAC1–4) are HPV positive while thorough HPV genotyping confirmed the absence of all assessed HPV sub-types in the *TP53* mutant line PMAC5. Importantly, STR analysis of the cell lines demonstrated that they are all independent cell lines and confirmed that each was derived from their respective parental tumour.

Using whole-exome sequencing we have characterised the genomic landscape of the panel. The genomic profile of each of the lines is consistent with the known genomic landscape of ASCC [17–20]. This includes a high prevalence of *PIK3CA* mutations and copy number gain at the *PIK3CA* locus in the HPV positive lines, and *TP53* mutations in the HPV negative line, both of which are the most frequently identified genomic aberrations in the respective ASCC sub-groups [5, 17–20]. The mutational load of the lines was also similar to that reported in the literature with PMAC1 at the high end of the range [18]. Consistent with a previous report [17], the trinucleotide signatures were dominated by an association with age (signature 1) and the HPV positive lines demonstrated the APOBEC signature associated with underlying viral infection and an increased frequency of *PIK3CA* mutations. We have also demonstrated the expression of MHC class I and PD-L1, both at baseline and following exposure to IFN-γ, across the panel of lines. PD-L1 upregulation was greatest with PMAC4 and least with PMAC1, both of which had CN gain and loss, respectively, at the JAK2 locus, which is implicated in the JAK/STAT pathway control of both MHC class I and PD-L1 membrane expression [42]. The high mutational burden combined with the low PD-L1 expression in PMAC1 may have contributed to the tumour’s excellent treatment response.

Importantly, the sensitivity of the lines to standard chemotherapeutic agents and radiotherapy is consistent with the clinical response observed in the patients from which they were derived. This includes the lines derived from relapse patients (PMAC4 and PMAC5) demonstrating resistance to both 5-FU and MMC, while the HPV negative cell line (PMAC5) demonstrates the greatest resistance to radiotherapy when compared to the viral positive lines. Interestingly, the finding that PMAC4, which was derived from a tumour that relapsed following chemoradiotherapy, is sensitive to a single fraction of 2 Gy irradiation, and even more so to higher single fraction doses, suggests that tumour cell-extrinsic factors may also play an important role in determining clinical responses to radiation therapy.

We also show that the panel of lines can be used to assess the sensitivity to a molecular targeted therapy. When treated with the PI3Kα inhibitor BYL719, four cell lines with mutation or copy number gain of *PIK3CA* demonstrated sensitivity, while PMAC2 was identified as being resistant despite harbouring both a *PIK3R1* mutation and a copy number gain at the *PIK3CA* locus. A possible explanation for this resistance is offered by the copy number gain of FGF3 and FGF10 (Fig. 5A) in this cell line, both of which have previously been defined as resistance mechanisms to BYL719 in breast cancer [43].

We have established and validated the first panel of human ASCC cell lines. These five new epithelial cell lines are tumourigenic and recapitulate many of the clinical and biological features of ASCC both in vitro and in vivo. As such, they fill a major gap that has hampered research into ASCC and we expect they will be a valuable resource for future biological and preclinical studies to develop our understanding of this rare disease and assess new therapeutic options.

## Methods

### Human tissue

Fresh tumour tissue (biopsy or resection tissue) from patients with histologically confirmed ASCC was collected from the operating theatre and washed three times with 50 ml of ice-cold RPMI tumour wash [RPMI 1640 + 2.5 mmol L-glutamine (Gibco, Thermo Fisher Scientific, Massachusetts) containing 100 U/ml Penicillin + 100 μg/ml Streptomycin (Gibco #15140122), Nystatin, 10 μg/ml (Sigma, Missouri), Gentamicin 10 μg/ml (Pfizer, New York City) and 0.05 μg/ml Amphotericin B (Gibco) over a period of 15 min. The fresh tumour tissue was macroscopically dissected with aseptic technique with representative pieces snap-frozen and stored at −80 °C for future genomic analysis, and formalin fixed and paraffin embedding for histopathological analysis and IHC. The remainder was used fresh for cell line development. In some cases where the amount of tissue was limited, the tumour was first expanded by implanting a small fragment of tumour intramuscularly in Nod-SCID-IL2Rγ^null^ (NSG) immunodeficient mice [44].

Written informed consent was obtained from all patients providing tissue and data for this study, which was performed in accordance with the declaration of Helsinki and approved by the Human Research Ethics Committee of the Peter MacCallum Cancer Centre (HREC/16/PMCC/100).

### Establishing cell lines

Tumour pieces (human or xenograft) were macroscopically dissected into a fine slurry with fragments measuring 0.5 mm (maximal dimension) or less. The resultant tumour aggregates were then transferred into a 24 well plate in 2 ml per well of RPMI 1640 containing 2.5 mM L-glutamine, 20% (v/v) foetal calf serum (FCS), 100 U/ml penicillin and 100 μg/ml streptomycin, and incubated at 37 °C in a 5% (v/v) CO_2_ incubator. The plate was initially not disturbed for a minimum of four days to facilitate adherence of the tumour explants to the plate, then half the media changed three times a week. Initially, fibroblast outgrowth occurred over a period of 7–10 days, after which epithelial cells grew out circumferentially from the tumour explant onto the bed of fibroblast cells. When the epithelial component occupied at least 70% of the well surface, the first passage was undertaken. Cells were detached with 1 ml/well TrypLE Express (Gibco), centrifuged and re-suspended in fresh RPMI 1640 containing 2.5 mM L-glutamine, 20% (v/v) FCS and 10 μM Y27632 (ROCK-inhibitor, Sigma) and re-seeded.

The time period between initial plating of the explants and first passage was up to 3 months and dependent upon the rate of epithelial cell expansion. The cells were passaged when they reached 70% confluency into progressively larger tissue culture plates and subsequently flasks, until the cell lines were robust (passage 10–15). Eradication of mouse fibroblasts was required following the establishment of a robust cell line from patient-derived tumour xenografts harvested from NSG mice. Multiple techniques were employed to assist with fibroblast elimination, including intermittent differential lifting with TrypLE Express, sequential FCS reduction to 10% and temporary exposure to L-glutamine free RMPI 1640 for 48–72 h. For T175 flasks, PMAC1, PMAC2 & PMAC3 were seeded at 5 million cells, PMAC4 at 1 million cells and PMAC5 at 2 million cells. All cell lines were maintained in RPMI 1640 containing 10% (v/v) FCS.

### Tumouroid cultures

Cell line-derived tumouroids were established by re-suspending the cells in ice-cold Matrigel and plated (one 40 μl drop) in a pre-warmed 24-well plate (Corning^TM^ Costar^®^, New York) at an optimal density of 5–50 × 10^2^ cells/40 μl. The plate was placed into a cell culture incubator at 37 °C and 5% CO_2_ for 30 min to allow polymerisation of each droplet, following which RPMI 1640 + 10% FCS was then added at 500 µl per well. The culture was replenished with fresh media every 3–4 days during tumouroid growth. Dense cultures with tumouroids ranging in size from 200 to 500 μm were passaged weekly. To passage, the tumouroids were collected in 1 ml of warm TrypLE Express using a P1000 pipette for fragmentation and disruption. Once small clusters and cells were obtained, digestion was halted by the addition of RPMI 1640 containing 10% FCS, with subsequent centrifugation and re-suspension in ice-cold Matrigel for replating. All cultures were screened for mycoplasma by PCR and confirmed negative before being utilised for experimental assays as outlined below.

### Peripheral blood mononucleocytes (PBMCs)

Blood was harvested from matched patients undergoing an ASCC biopsy, with between 15 and 30 ml collected in EDTA tubes for processing to obtain PBMCs. Density gradient SepMateTM-50 tubes (Stemcell Technologies, Vancouver, BC, Canada) containing Ficoll-Paque PLUS (GE Healthcare Life Sciences, Mississauga, ON, Canada) were used with blood diluted 1:2 with phosphate-buffered saline (PBS), centrifuged at 1200 × *g* for 10 min and the mononuclear cells in the top layer collected, washed (PBS) and re-centrifuged. An ammonium-chloride-potassium (ACK) lysis was undertaken with 3 ml of ACK lysis buffer (Gibco, Thermo Fisher Scientific) for 3 min to remove residual red blood cells before the cells were washed, centrifuged and cryopreserved for downstream genomic analysis.

### Light microscopy

Cell morphology was assessed using the AMG EVOS FL (Advanced Microscopy Group) phase-contrast microscope.

### Haematoxylin & eosin staining + immunohisto-/cyto-chemistry

Tissues were fixed in 10% neutral buffered formalin for 24–48 h and then embedded in paraffin. Tumouroids and cells were fixed in 4% paraformaldehyde, with the tumouroids embedded in Histogel^TM^ (Thermo Fisher Scientific). Sections were stained with haematoxylin and eosin as well as IHC. For IHC, positive and negative controls, including a slide where the primary antibody was omitted, were processed with the study samples to confirm appropriate antibody specificity. The selected tumour Superfrost^TM^ Plus slides (Thermo Fisher Scientific) were baked at 60 °C for 45–60 min to melt the paraffin. The slides were then dewaxed and rehydrated in the Leica Jung Autostainer (CAHM at PMCC) utilising the dewax protocol. Antigen retrieval was performed by placing the slides in an alkaline (EDTA, 1 mM, pH 8) or acidic buffer (Citrate, 10 mM, pH 6) in a Dako pressure cooker at 125 °C for 3 min, and then allowed to cool to 90 °C for 10 s. The slides were cooled for a further 20 min before washing with MilliQ H_2_O. Slides were then rinsed in tris-buffered saline with 0.05% (v/v) Tween (TBS-T, pH 7.6). A Pap pen was used to circle the tumour section on the slide.

For immunocytochemistry, cells were lifted with TrypLE^TM^ Express from tissue culture flasks seeded a day prior. After washing with RPMI 1640 + 10% FCS and centrifugation, the cells were resuspended in a 15 ml Falcon tube, and counted to allow appropriate seeding into an 8-well Nunc^TM^ Lab-Tek^TM^ II Chamber slide^TM^ (Thermo Fisher Scientific). Each chamber was filled with a total of 300 μl media. The chamber slide was then transferred to a 37 °C 5% CO_2_ incubator to allow the cells to settle and adhere overnight. The following day, ensuring all cells were appropriately 40–70% confluent, the media was aspirated, and the wells were washed with 500 μl PBS. After aspiration of the PBS, gentle fixation was performed with 500 μl 4% paraformaldehyde (PFA, Thermo Fisher Scientific) for 20 min at room temperature. The PFA was then removed, with a further 2× washes with 500 μl PBS. Permeabilisation was then undertaken with 500 μl of 0.2% TritonX for 10 min at room temperature. Two washes with 0.05% TBS-T were performed, following which the immunocytochemistry staining was undertaken.

Superfrost^TM^ Plus & Chamber Slides were processed in a similar fashion, with the exception of reagents being added to each well of the chamber slide in contrast to the Pap marked area or whole slide. Endogenous peroxidase blocking was undertaken with a quenching step of 3% H_2_O_2_, at room temperature for 10 min on a rocker. A further 2 × 5-min washes with 0.05% TBST were then performed on a rocker. Further blocking was then undertaken with 10% (w/v) bovine serum albumin for 1 h in a humidified chamber at room temperature to reduce non-specific binding. A further 2 × 5-min washes were then undertaken with 0.05% TBST on a rocker. Following the completion of the blocking steps, the primary antibody was added at an optimised concentration. The slides were then incubated in a humidified chamber at 4 °C overnight. The details of the antibodies used for IHC are provided in Supplementary Table 5.

The following day, slides were washed 3 times with 0.05% TBS-T on a rocker for 5 min. The appropriate secondary antibody was then added to the Pap pen marked sections and incubated in a humidified chamber for 30 min at room temperature. A further series of 3× washes was undertaken with 0.05% TBS-T on the rocker for 5 min. The slides were then developed with the Envision Plus Dako System, beginning with the positive control to determine the duration of exposure for subsequent slides. Slides were then placed in MQ H_2_O, before being counterstained with haematoxylin and sequentially dehydrated in ethanol and placed in histolene by utilising the Leica Jung Autostainer. MM24 (Leica Biosystems) mountant was then applied and the slides cover slipped.

### HPV Assessment

Assessment of HPV subtypes present in the cell lines was undertaken via a collaboration with the VCS Foundation. Five PCR based assays targeting either the viral L1 (cobas^®^ 4800 HPV & cobas^®^ HPV (Roche, Pleasanton, California), Anyplex^TM^ II HPV HR (Seegene, Seoul, South Korea)) or E6/7 regions (BD Onclarity^TM^ HPV Assay (BD Diagnostics, Sparks, Maryland), Xpert^®^ HPV Test (Cepheid, Sunnyvale, California)) were utilised to assess for HPV 16, 18 and 12 other high risk genotypes (31, 33, 35, 39, 45, 51, 52, 56, 58, 59, 66, 68). The Linear Array^®^ HPV Genotyping assay (Roche) assessed for a total of 37 genotypes, including 13 high risk (16, 18, 31, 33, 35, 39, 45, 51, 52, 56, 58, 59, 68) and 24 low risk (6, 11, 26, 40, 42, 53, 54, 55, 61, 62, 64, 66, 67, 69, 70, 71, 72, 73 (MM9), 81, 82 (MM4), 83 (MM7), 84 (MM8), IS39, CP6108) subtypes.

### Mycoplasma assessment

Mycoplasma was assessed for via PCR on cell line DNA derived from cells that had been in culture for a minimum of 2 days. DNA was extracted using QuickExtract^TM^ (QE) DNA Extraction Solution (Lucigen, Middleton, Wisconsin). The primers are listed in Supplementary Table 6. The PCR reaction was performed in the presence of 5 μl of 5× MangoTaq Buffer + 1.5 μl 25 mM MgCl_2_ + 0.5 μl 10 mM dNTPs + 2 μl Myco Fwd + Rev Primers + 0.5 μl Cyto Fwd + Rev Primers + 14.25 μl MQ H_2_O + 0.25 μl Mango Taq 5 U/μl by the thermocycler and involved 95 °C for 5 min followed by 45 cycles of: 95 °C for 30 s, 53 °C for 30 s and 72 °C for 30 s. This was followed by one cycle of 72 °C for 5 min and 14 °C hold. The samples were run on a 1.5% agarose gel with Midori Green Advance (Nippon Genetics #MG04) and imaged with the Gel Doc^TM^ XR Imager (BioRad). Cytochrome B was used as the loading control, to confirm the presence of gDNA in each lane with this band identified at 375 bp. The mycoplasma band was at 520 bp, with a 1 kb ladder used as the reference. All samples were assessed in triplicate.

### Seeding (plating) efficiency

The plating efficiency of each cell line was determined to identify the optimal density for passaging each cell line. This was performed by seeding cells into six-well plates in triplicate at three different cell densities (PMAC1–2/4/8 × 10^3^; PMAC2–10/30/60 × 10^3^: PMAC3–1/2/4 × 10^3^; PMAC4/PMAC5–0.2/0.5/1 × 10^3^), and the plates incubated with regular media changes out to 7 days. The media was then aspirated and the plates washed with PBS before the colonies were fixed and stained with 3 ml of 0.5% w/v crystal violet in 25% methanol for 5 min, rinsed with water and allowed to air dry. Discrete colonies of ≥30 cells were manually counted under an inverted microscope and divided by the total number of cells seeded and averaged across the three cell densities to determine the plating efficiency.

### Cellular proliferation assay

Cell proliferation and migration was assessed using the xCELLigence RTCA-DP (ACEA biosciences, Noble Park North, Australia) E-plate platform which uses non-invasive electrical impedance monitoring to quantify cell growth and proliferation. The proliferation protocol as published by ACEA biosciences was followed strictly. Following equilibration and assessment of the background impedance with media alone, cells were seeded into the wells of an E-plate in duplicate and mounted in the xCELLigence RTCA platform within an incubator at 37 °C and 5% CO_2_. The increased contact of cells across the plate increases the impedance to current flow, which is converted into a real time surrogate measure of cellular proliferation by the xCELLigence software, designated the cell index. The E-plate has a 2 × 8 flat bottom well configuration. Seeding densities for each cell line were 2 × 10^4^ (PMAC1, PMAC2, PMAC3), 1 × 10^4^ (PMAC4, PMAC5). Time points were set at 15 min for the first 6 h followed by hourly out to 150 h.

The initial 12–24 h of the curve relates to the settling, adherence, flattening and migration of the squamous cells prior to undergoing proliferation. Consequently, all of these events are associated with greater electrode contact and therefore increased resistance, leading to a non-growth phase related increase in the cell index. Following this period, the cells begin to proliferate at variable rates dependent on their doubling time and represented by the exponential aspect of the curve. The final plateau relates to either cell confluence or exhaustion of the media. The exponential growth equation [*y* *=* y0*exp(k*x)] (where *y0* is the value when *x* is zero, *k* is the rate constant (inverse minutes) was applied to the growth phase of the curves (Supplementary Fig. 3C) to allow calculation of *k* and then subsequently using the equation [*ln(2)/k*] to give the doubling time.

### CD3 Tumour infiltrating lymphocyte assessment

Under light microscopy, four 250 μm^2^ areas were assessed in hot-spot regions of high (Tumour Infiltrating Lymphocytes (TIL) infiltrate which included stroma and tumour, using a 0.5 mm × 0.5 mm grid with the ×20 objective magnification. The average of the four counts was the representative CD3 TIL count.

### Cellular migration assay

The Cell Invasion Migration (CIM) plate was used for undertaking the migration assay. This plate differs from the E-plate in having both an upper and lower chamber, with electrodes located on the underside of the microporous membrane, which is a component of the upper chamber. The migration protocol as published by ACEA biosciences was followed strictly. RPMI + 20% FCS was added to the bottom chamber well as a chemoattractant in both assays. Cells were seeded in RPMI without FCS into the upper chamber at densities of 8 × 10^5^ (PMAC1, PMAC2, PMAC3) and 4 × 10^5^ (PMAC4, PMAC5). Measurements were made at 15 min intervals for a period of 24 h. The experiment was abbreviated at this point, in order to remove the potential of proliferation contributing to the cell index reading.

Modelling with linear regression after removal of the first 3 h (experimental setup, cell settling and adherence) of the assay, allowed the inverse gradient of the resultant lines to be calculated as a surrogate of each cell lines’ migration potential (Supplementary Fig. 1D).

### IFN-γ Stimulation and modulation of MHC class I and PD-L1

Modulation of the immune relevant cellular markers, MHC class I and PD-L1 were assessed at baseline and following exposure to IFN-γ. Cell were seeded in a 12 well plate (PMAC 1, 2, 3–2 × 10^5^, PMAC 4, 5–1 × 10^5^) in triplicate for each condition (baseline, stimulation) with 1.5 ml of RPMI-1640 + 10% FCS and allowed to adhere overnight. The following day, media was removed and replaced with fresh media alone or media with 100 ng/ml IFN-γ (BD Pharmingen #554617). The plates were returned to the 37 °C 5% CO_2_ incubator for 48 h. The plates were then assessed under an inverted microscope for the effect on the cell line colony size (qualitative) and number (quantitative). Cells were then lifted with TrypLE Express and washed with PBS, pelleted and transferred to a 96 well plate for FACS staining with anti-PD-L1 and anti-MHC class I antibodies as detailed below. Flow cytometric assessment was undertaken with the LSRFortessa platform, with the Mean Fluorescent Intensity (MFI) used as a marker across the isotype, control and stimulation conditions. Data were analysed with Graphpad Prism, assessing the log-fold change in the MFI.

The IFN-γ stimulation was also undertaken on cell line tumouroids in a similar fashion. Established cell line tumouroids (>50 μm) were plated (>10/well) without disruption in fresh matrigel in a 24-well plate with 1 ml of RPMI-1640 + 10% FCS and returned to the incubator overnight. The media was replaced the following day with fresh media alone or media supplemented with 100 ng/ml IFN-γ and the plates returned to the incubator for 48 h. The cell line tumouroids were then retrieved, fixed with 4% PFA and washed before embedding in Histogel^TM^. Sections were then stained with immunohistochemistry for anti-MHC class I (Abcam, #ab70328 EMR8-5; EDTA 80 Buffer) and anti-PD-L1 (Ventana, SP263, #790-4905; EDTA Buffer). The parent tumour tissue was also stained by IHC for PD-L1 for comparison. Representative images were taken with the Olympus BX61 for assessment of PD-L1 expression based on the Tumour Proportion Score (TPS), as <1%, 1–49% and ≥50% as previously defined [45].

### Tumour and cell line xenografts

Patient-derived xenografts (PDX) were used to expand patient biopsy material when patient tissue was limited. PDXs were established by implanting small fragments of fresh patient biopsy tissue intramuscularly on the dorsum of NSG mice, as previously described [44]. To assess the tumourigenic potential of the cell line, 5 million cells suspended in 100 µl of 1:1 phosphate-buffered saline and Matrigel (Corning) were subcutaneously injected into the flank of 6-week old female nude or NOD-SCID IL-2Rγ^null^ (NSG) mice [46]. Five mice per line were utilized to allow adequate tumourigenicity and growth kinetic assessment. Tumour volume was assessed with caliper measurements every 3–4 days, and calculated using the formula (length × width^2^)/2. All mice were euthanised when tumours reached 1500 mm^3^ (or earlier if showing signs of discomfort or distress). All animal experiments were undertaken in accordance with the National Health and Medical Research Council *Australian Code for the Care and Use of Animals for Scientific Purposes* (8^th^ Edition, 2013) with ethical approval obtained from the Peter MacCallum Cancer Centre Animal Experimentation and Ethics Committee (PMCC AEEC-E601).

### Scanning electron microscopy

Cell line tumouroids were plated and maintained in a 24 well plate as outlined above, until established at 7–10 days. The tumouroids were retrieved from the matrigel without significant disruption utilising Cell Recovery Solution (Corning^TM^). The tumouroids were then plated in RPMI 1640 + 10% FCS onto a purpose cut segment of thin aclar^®^ plastic film (Electron Microscopy Sciences, Hatfield, Pennsylvania), which lay at the base of the well in a 24 well plate. The aclar^®^ plastic was sterilised with 90% ethanol prior and allowed to dry before being placed with a sterile technique into the well. The plate was returned to the incubator and allowed to sit for 48 h during which time the tumouroids adhered to the aclar^®^ plastic. The media was then removed and the tumouroids fixed in 2% PFA, 2.5% glutaraldehyde in 0.1 sodium cacodylate buffer (Thermo Fisher Scientific) at 4 °C. The tumouroids were then washed in 0.1 M sodium cacodylate buffer before being stored in 0.08 M sodium cacodylate buffer (Electron Microscopy Sciences) with 5% sucrose at 4 °C.

The Leica EM CPD300 critical point drier was used to dehydrate tissue samples. The tumouroids adherent to the aclar^®^ plastic were then mounted on a scanning electron microscopy stub (agar scientific) and gold sputter coated with an Emscope SC500 before being imaged with the Jeol JCM-6000PLUS Neoscope Benchtop scanning electron microscope. Images were acquired at 10 kV under high vacuum.

### DNA Isolation

Genomic DNA was extracted from the cell lines, PBMCs and tumour tissue using the Qiagen DNeasy Blood and Tissue kit according to manufacturer specifications. DNA concentration was measured using Nanodrop and Qubit (Thermo Fisher Scientific).

### Short tandem repeat (STR) analysis

STR analysis was undertaken on the panel of cell lines and compared with genomic DNA extracted from the parent tumour tissue from which the cell line was derived. Comparison was made across 10 loci, as recommended by ATCC (American Type Culture Collection).

### Whole-exome sequencing

Whole-exome sequencing analysis was performed on paired DNA samples of cell line and matched normal (PBMCs) and also corresponding parent tumour and matched normal (PBMCs). The process utilised Unique Molecular Identifiers (UMIs) to provide error correction and increased accuracy. Capture of the coding sequences from individual libraries for each sample was performed using the SureSelect Whole Exome Sequencing Kit v6 (Agilent Technologies). Sequencing of the captured libraries was then performed using the Novaseq 6000 Genome Analyser (Illumina) with a mean target coverage of 124X achieved and a mean fragment length of 225 bp. Duplicated reads were handled by our bioinformatics pipeline using Picard UmiAwareMarkDuplicatesWithMATECigar (v2.17.3, http://broadinstitute.github.io/picard). Quality control was performed with FastQC (v0.11.7, Babraham Institute) and the sequenced reads were aligned to the GRCh37 human reference genome using Burrows-Wheeler Aligner (BWA, v0.6.2) [47–49]. Raw genomic data is currently available from the European Genome-Phenome Archive (Accession number EGAD00001007001).

### Detection of somatic mutations and mutational load

Quality filters were applied to the samples for the selection of both single nucleotide variants (SNVs) and small insertions/deletions, predicted as present in the cell lines/tumours and not in their matched PBMC’s (normal control). The variants that were included met all of the following quality criteria: (1) two independent callers for variants from MuTect, MuTect2 (v4.0.8.0) [50] and VarDict [51]; (2) gnomad population allele frequency missing or <1%; (3) on the canonical transcript; (4) with a total read depth of 10 or more in the samples, with at least 5 supporting reads (allele depth 5) coming from both forward and backward strands. This pipeline adopted GATK Best Practices for variant-calling by performing base quality score recalibration of the deduplicated reads with GATK tools [52].

The mutational load of the cohort of ASCC lines was compared to other TCGA (The Cancer Genome Atlas, https://www.cancer.gov/tcga) listed cohorts and generated by Maftools (Bioconductor). The R-library upSet was used to identify the overlap of shared variants between cell lines and parent tumour.

### Detection of mutational trinucleotide signatures

To generate the mutational trinucleotide signature plots for each sample, the computational framework of Alexandrov et al. [25] and the R Package deconstructSigs (R Core Team, 2015) was utilised [53]. An iterative approach was employed to determine the weights to assign to each signature, with the raw trinucleotide counts normalised by the number of times each trinucleotide context is observed in the human exome. The mutation signatures (v2, 2015) are derived from the COSMIC database [54].

### Detection of somatic copy number alteration

The somatic copy number alterations in the parent tumour and in the cell lines compared to the matched PBMC’s were characterised using FACETs (v0.5.0) [55]. The copy number state of a gene was assigned based on the copy number state of the genome segment containing it. Less than 1% of genes were not assessable because they overlapped with more than one genome segment or did not fall into any segment. The copy number frequency plots were generated for all genes, while the cytoband copy number plot was constructed by segmenting the genome into bins of 10Mbp size, and each bin/box coloured by integer copy number.

### Flow cytometry

Surface staining following the IFN-γ stimulation, utilised a 96-well v-bottom plate (Nunc^TM^, Thermo Fisher Scientific). This included an unstained sample and Fixable Viability Stain (FVS) sample, with 0.5 × 10^6^ cells added to respective wells. For the isotype control and stained samples (IFN-γ +/− for each cell line), 1 × 10^6^ cells were added. The viability stain was added to FVS and isotype controls and the stained sample, with the unstained control resuspended in PBS. Cells were incubated at room temperature for 10 min in the dark. The plate was then centrifuged at 400 × *g* for 4 min at room temperature, supernatant discarded, and the cells washed with PBS. Cells were resuspended in 50 μl of Human Fc Block (BD Pharmingen^TM^) diluted 1:10 in Staining Media (SM; RPMI 1640, 4% FCS, 100 U/ml penicillin and 100 μg/ml streptomycin). After 10 min incubation in the dark at 4 °C, 100 μl antibody mix (Human HLA Class I A,B,C PE BD Biosciences, W6/32 mouse; PD-L1 (CD274)-APC-R700 BD Biosciences, Mouse) and corresponding isotype control antibodies were added to the stain and isotype control samples. SM was added to unstained and FVS controls and samples incubated for 30 min at 4 °C in the dark. Samples were then washed twice with SM and fixed with 100 μl 1% PFA in SM. Cells were then resuspended in 200 μl SM and transferred to FACS tubes and acquired on the BD LSRFortessa^TM^ X-20 within 48 h. Samples were stored at 4^o^C protected from light during this period until acquired. FACS data analysis was performed using FlowJo software (BD).

### Drug cytotoxicity and cell viability assays

Cells were seeded in a flat bottom 96 well plate 24 h prior to treatment. They were seeded at variable densities depending on the cell line used, in a volume of 200 μl per well, with all experimental conditions performed in triplicate and on two separate occasions. Cytotoxic drugs were assessed across 10 concentrations including a negative control of media alone. After 24 h, media was aspirated, and the appropriate drug concentration, vehicle control or media alone added, and the plate returned to the incubator for 96 h.

Following the 96 h incubation, a Resazurin (AlamarBlue^®^) assay was undertaken to assess cell viability as an inverse marker of cytotoxicity. This assay quantifies the conversion of non-fluorescent resazurin (Sigma #R7017-1G) to fluorescent resorufin by mitochondrial metabolic activity. To each well, 20 μl of 20% (v/v) Resazurin solution was added without removing pre-existing media and the plate returned to the incubator for 2–6 h. A 20% (v/v) Resazurin (AlamarBlue^®^) solution is produced by dissolving sequentially 75 mg of Resazurin, 12.5 mg of Methylene Blue (Sigma #MB-1), 164.5 mg of potassium hexacyanoferrate (III) (Sigma #P8131) and 211 mg of potassium hexacyanoferrate (II) trihydrate (Sigma #P9387) in 500 ml of sterile PBS within the confines of a Tissue Culture Hood.

A FLUOstar OPTIMA microplate reader (BMG labtech, Mornington, Australia) was used for fluorescence measurement, with an excitation of 540 nm and an emission of 590 nm. The percentage of viable cells was calculated as follows: $\left( {{{{\bar{\mathrm x}}}}_{{{\mathrm{D}}}} - {{{\bar{\mathrm x}}}}_{{{\mathrm{M}}}}} \right/\left( {{{{\bar{\mathrm x}}}}_{{{\mathrm{V}}}} - {{{\bar{\mathrm x}}}}_{{{\mathrm{M}}}}} \right) \times 100$, where ${{{\bar{\mathrm x}}}}_{{{\mathrm{D}}}}$: mean fluorescence of drug-treated wells, ${{{\bar{\mathrm x}}}}_{{{\mathrm{M}}}}$: mean fluorescence of media only wells, and ${{{\bar{\mathrm x}}}}_{{{\mathrm{V}}}}$: mean fluorescence of vehicle-treated control wells. Dose-response curves and GI50 values were generated using Graphpad Prism.

### Radiotherapy clonogenic survival

To investigate the effects of radiotherapy on the clonogenic survival of the cell lines, cells were seeded in 6 well plates and allowed to adhere for 12 h before treatment with radiotherapy doses of 2, 4, 6, 8 or 10 Gray (Gy). The seeding density was optimised and dependent on the radiotherapy dose being delivered as outlined in Supplementary Table 7 for each cell line. The optimisation was undertaken by plating out a minimum of three cell densities for each line and radiotherapy dose. The density that resulted in a colony count of 25–150 was utilised to determine the surviving fraction. Cell colonies were fixed with 3 ml of 0.5% w/v crystal violet in 25% methanol for 5 min, rinsed with water and allowed to air-dry. Discrete colonies of ≥50 cells were counted manually under an inverted microscope with the aid of a grid. The colony count was expressed as a percentage of the untreated group, using the following formula: Surviving fraction = Number of colonies formed/(Number of cells seeded × Plating efficiency), where plating efficiency was the surviving fraction for 0 Gy. Graphpad Prism (San Diego, CA 92108, USA) was used to fit the linear quadratic equation [SF(D) = exp(−αD − βD^2^)] to the data using non-linear regression with 1/Y^2^ weighting.

All authors had access to the study data and have reviewed and approved the final manuscript.

## Supplementary information

Supplementary Figure Legends

Supplementary Figure 1

Supplementary Figure 2

Supplementary Figure 3

Supplementary Figure 4

Supplementary Tables

## Data Availability

The datasets generated and analysed in this study are available from the corresponding author on reasonable request. Raw genomic data is available from the European Genome-Phenome Archive (EGA), with accession number EGAD00001007001.
