# Supplementary material for: Molecular and genomic characterisation of a panel of human anal cancer cell lines
Source: Cell Death Dis. 2021 Oct 18;12(11):959. doi: 10.1038/s41419-021-04141-5 (PMC8523722; doi:10.1038/s41419-021-04141-5)
Supplement: Supplementary file 1 — Supplementary Figure Legends [file 41419_2021_4141_MOESM1_ESM.docx]

**Supplementary Figure 1. ASCC Cell Line Establishment and Culture Characteristics**

A. Epithelial cell outgrowth from tumour explants. Brightfield photomicrographs from left to right demonstrate initial tumour explant (indicated by dotted line) adherence with fibroblast outgrowth, followed by squamous cell expansion (indicated by dashed line) onto the fibroblast bed over a period of 3 weeks (left images – day 7, middle images – day 14, right images – day 21). (upper row – x 10 image, scale bar 400μm, lower row – x 20 image, scale bar 200μm)

B. Mycoplasma Assessment by PCR. PCR for mycoplasma (520bp) and cytochrome C (375bp; loading control) run on 1.5% agarose gel containing Midori Green utilising gDNA extracted from cell line pellets. (P1, PMAC1; P2, PMAC2; P3, PMAC3; P4, PMAC4; P5, PMAC5; +ve – mycoplasma positive control; –ve – mycoplasma negative control; H_2_O – DNA negative control; LAD – 100bp ladder).

**C.** The exponential growth equation (solid lines) was applied to the exponential growth phase of the real-time curves (***Figure 1C,*** 24 - 96 hours – dotted lines), demonstrating close alignment with the real time data.

**D.** Modelling with linear regression (solid line) after removal of the first 3 hours (experimental setup, cell settling and adherence) of the migration assay (***Figure 1D***) demonstrates close alignment with the real time data (dotted line).

**Supplementary Figure 2. Whole Exome Sequencing derived Circos Plots for each ASCC Cell Line and Matched Parent Tumours**

Circos plots demonstrating the single nucleotide variants (SNVs - blue) and small insertion or deletions (Indel - red) for the ASCC cell lines (Outer track, PMAC1-5) and parent tumour (inner track, PMAC2, 3, 5).

**Supplementary Figure 3. Somatic Copy Number Analysis for each ASCC Cell Line and Matched Parent Tumours**

Copy number analysis for PMAC1, PMAC2, PMAC3, PMAC4, and PMAC5 cell lines and matched parental tumours. The images display the copy number variation for each sample, with the minor copy number (red line) indicating the number of copies of the least frequent allele, and the total copy number (black line) the sum of the major and minor allele counts. The cellular fraction harbouring the copy number variation is displayed in the bottom bar (tan – normal (diploid), shades of blue – estimated fraction; cf – cellular fraction, em – estimated mean). The top image for each line represents the cell line SCNA and the bottom image the matched parental tumour SCNA. This demonstrates that the cell lines had a greater variation in the SCNA than the matched parent tumour. For PMAC2, PMAC3 and PMAC5, there was significant overlap in the SCNA between the cell line and the parent tumour.

**Supplementary Figure 4. Mutational Trinucleotide Signatures in the ASCC Cell Lines**

The mutational trinucleotide signatures for **A.** PMAC1, **B.** PMAC2, **C.** PMAC3, **D.** PMAC4, and **E.** PMAC5 cell lines as determined from whole exome sequencing analysis. The pie charts demonstrate the relative predominance of mutational signatures present in each cell line based on the fraction of each mutation type demonstrated in the bar graph.^26^
