# Supplementary figures and images for: Molecular and genomic characterisation of a panel of human anal cancer cell lines
Source: Cell Death Dis. 2021 Oct 18;12(11):959. doi: 10.1038/s41419-021-04141-5 (PMC8523722; doi:10.1038/s41419-021-04141-5)

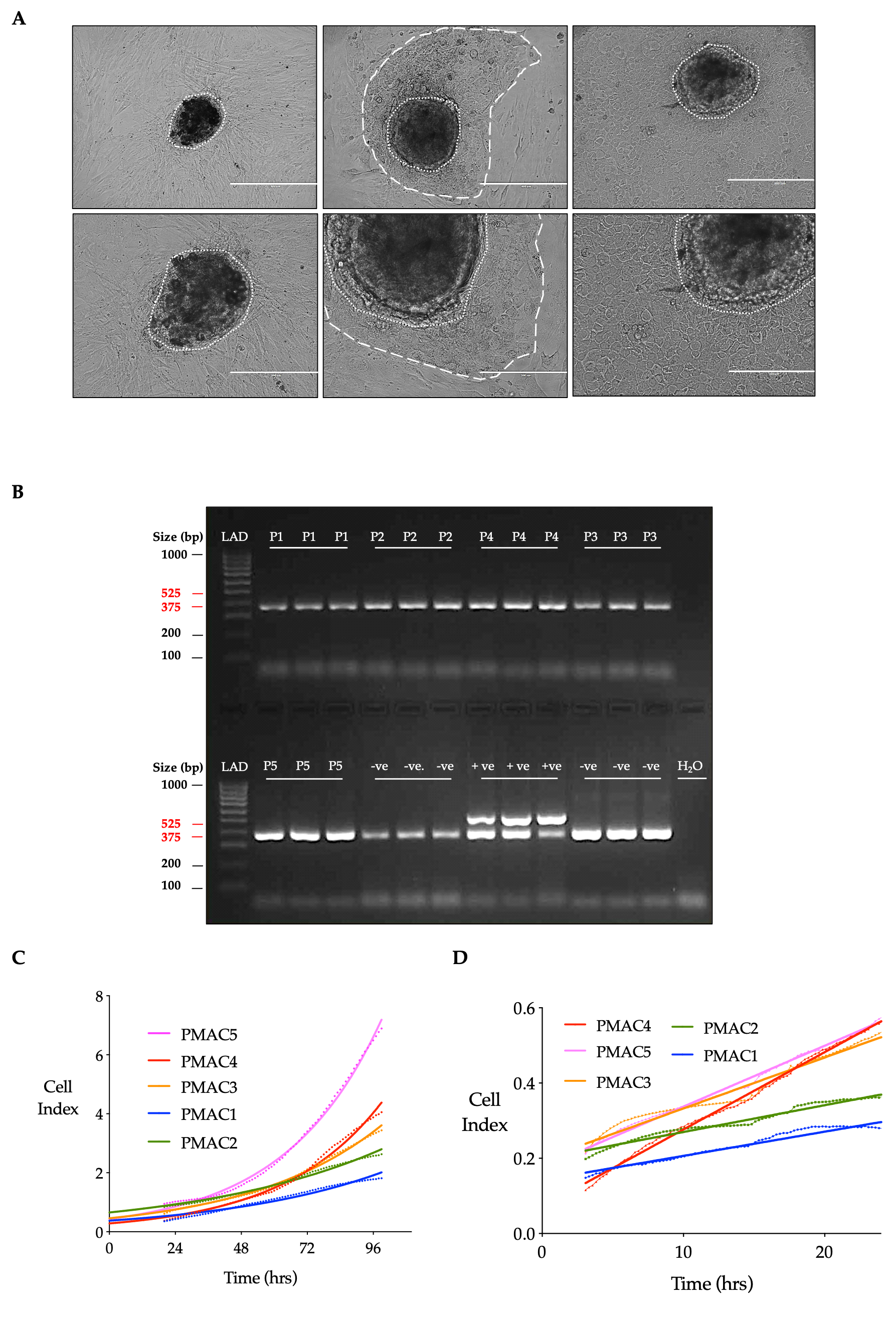

Supplement: Supplementary file 2 — Supplementary Figure 1 [file 41419_2021_4141_MOESM2_ESM.png]

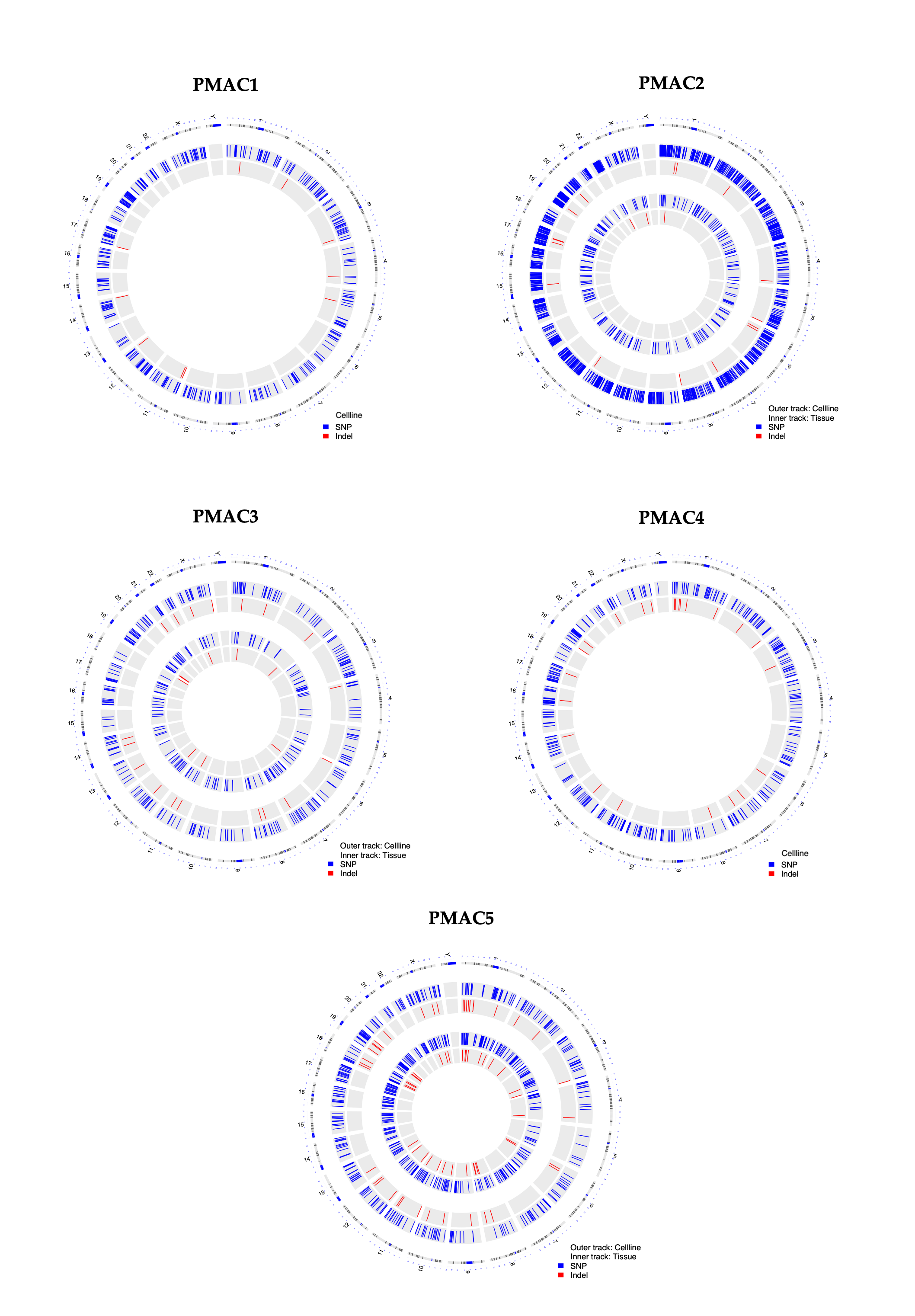

Supplement: Supplementary file 3 — Supplementary Figure 2 [file 41419_2021_4141_MOESM3_ESM.png]

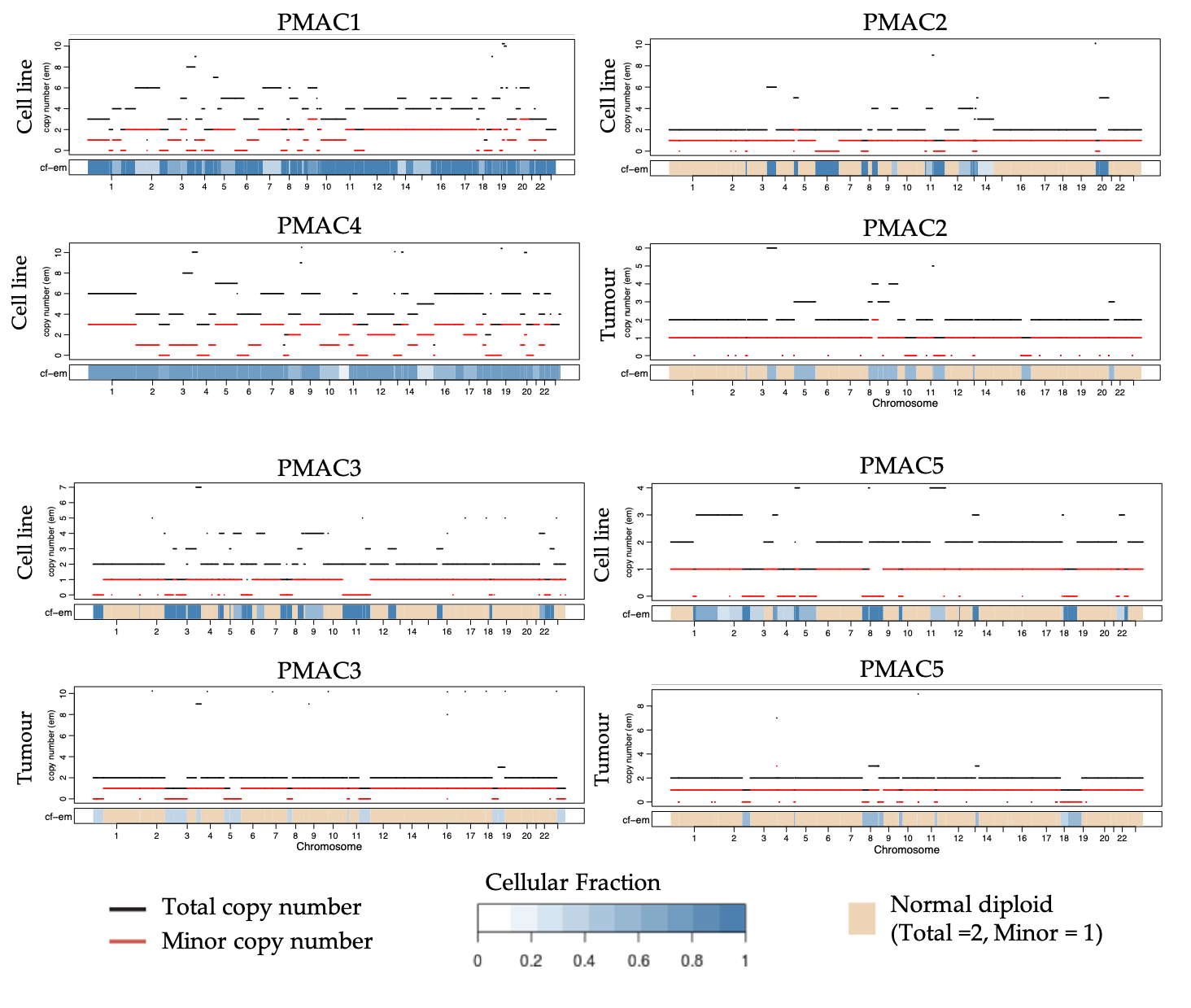

Supplement: Supplementary file 4 — Supplementary Figure 3 [file 41419_2021_4141_MOESM4_ESM.png]

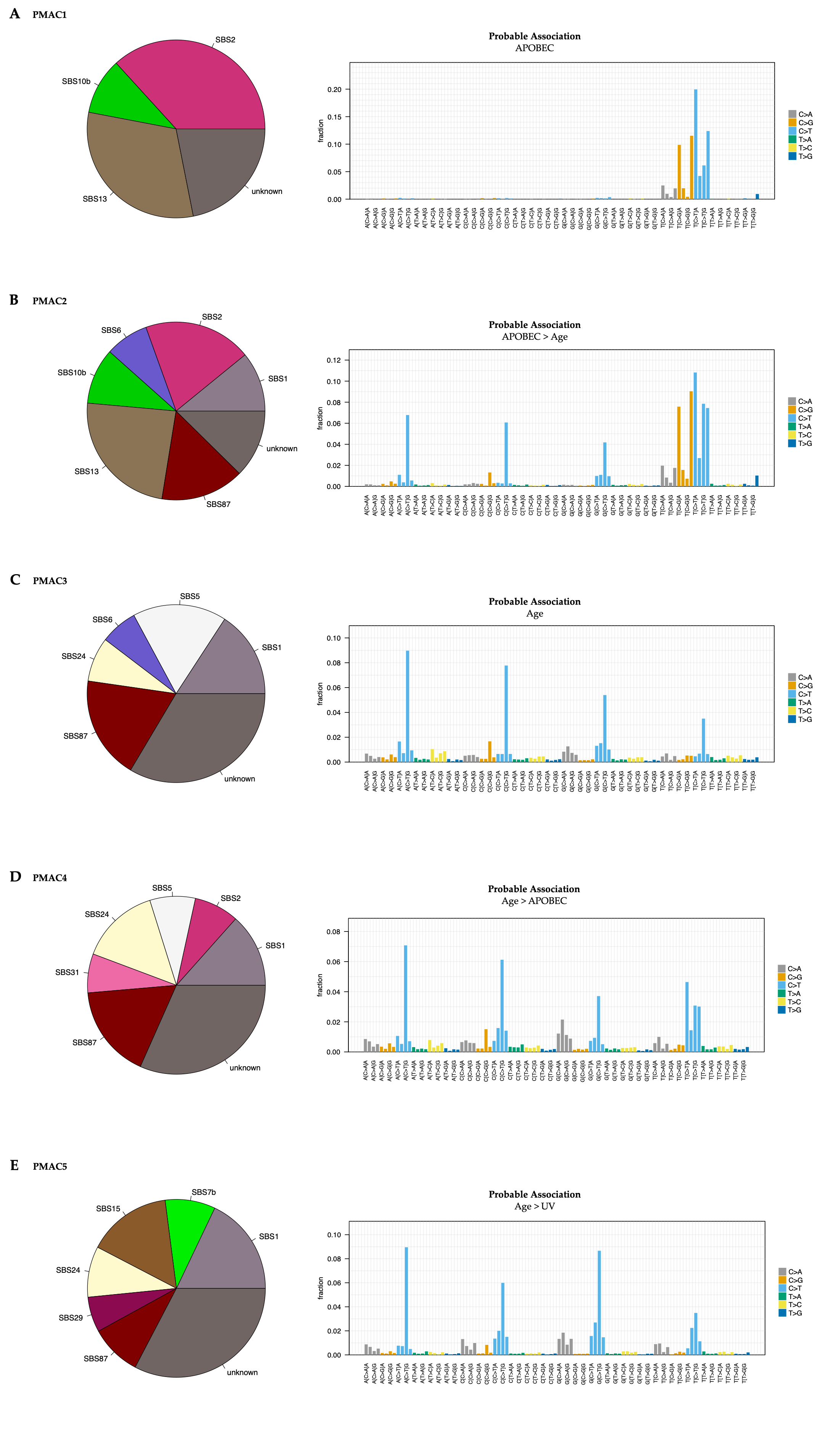

Supplement: Supplementary file 5 — Supplementary Figure 4 [file 41419_2021_4141_MOESM5_ESM.png]
