# Supplementary material for: Molecular and genomic characterisation of a panel of human anal cancer cell lines
Source: Cell Death Dis. 2021 Oct 18;12(11):959. doi: 10.1038/s41419-021-04141-5 (PMC8523722; doi:10.1038/s41419-021-04141-5)
Supplement: Supplementary file 6 — Supplementary Tables [file 41419_2021_4141_MOESM6_ESM.docx]

| **Patient** | **Age / Sex**  **Supplementary Table 1 Success of Establishing ASCC Preclinical Models** | **Location** | **Differentiation** | **Stage** | **Tumour** | **Treatment** | **Response / Outcome** | **1^0^ Xenograft** | **Cell Line** |
| --- | --- | --- | --- | --- | --- | --- | --- | --- | --- |
| 1 | 58 M | Canal | Mod | T2N0 (IIA) | Primary | 5FU / MMC | ANED 5 years | Yes | Yes (PMAC1, XG) |
| 2 | 45 F | Canal | Mod | T2N1 (IIIA) | Primary | 5FU / MMC | ANED 7 years | Yes | Yes (PMAC2, XG) |
| 3 | 46 F | Canal | Poor | T3N1 (IIIC) | Primary | 5FU / MMC | ANED 5 years | Yes | Yes (PMAC3, XG) |
| 4 | 62 F | Margin | Poor | T2N0 (IIA) | Primary | 5FU / MMC | ANED 5 years | No | *N/A* |
| 5 | 62 F | S/C Node | Mod | T2N1M1 (IV) | Metastatic | 5FU / MMC;  CTx + RTx, VATs | ANED 2 years | No | *N/A* |
| 6 | 56 M | Canal | Mod | T2N1 (IIIA) | Primary | 5FU / MMC | ANED 3 years | No | *N/A* |
| 7 | 47 F | Canal | Poor | T4N1 (IIIC) | Primary | 5FU / MMC  Pall CTx | LR + D Failure  AWD 2 years | No | *N/A* |
| 8 | 62 F | Canal | Mod | T4N1 (IIIC) | Primary | 5FU / MMC | ANED 2 years | Yes | No (XG) |
| 9 | 61 F | Canal | Mod | T3N0 (IIB) | Primary | 5FU / MMC | ANED 2 years | No | *N/A* |
| 10 | 46 M | Canal | Mod to Poor | T3N1 (IIIC) | Local Relapse | 5FU / MMC  Pall CTx | LR + D Failure  Died 20 months | Yes | Yes (PMAC4**,** 1^0^) |
| 11 | 69 F | Canal | Mod | T2N0 (IIA) | Primary | 5FU / MMC | ANED 1 year | No | *N/A* |
| 12 | 51 F | Canal | Mod to Poor | T1N0 (I) | Local Relapse | 5FU / MMC  APR | LR Failure  Died 2 years | Yes | Yes (PMAC5**,**  1^0^) |
| 13 | 77 M | Canal | Mod | T3N0 (IIB) | Local Relapse | 5FU / MMC  Pall RTx | LR Failure  Died 18 months | No | No (1^o^) |
| 14 | 60 F | Canal | Well | T4N1 (IIIC) | Primary | 5FU / MMC | ANED 2 years | No | *N/A* |
| 15 | 67 F | Canal | Mod to Poor | T3N1 (IIIC) | Local Relapse | 5FU / MMC | LR Failure  Died | Yes | No (1^0^, XG) |

*N/A – Not Applicable; (Name of Cell Line); XG - Cell line attempt from xenograft tissue; 1^0 -^ Cell line attempt from primary tissue; ANED – Alive No Evidence of Disease; LR – Locoregional; D – Distant; AWD – Alive With Disease; 5FU – 5-Fluorouracil; MMC – Mitomycin C, RTx - Radiotherapy; VATs – Video Assisted Thoracoscopic Surgery; Pall RTx – Palliative Radiotherapy*

**Supplementary Table 2 STR Analysis of ASCC Cell Lines and Parental Tumour Samples**

| **Loci** | **PMAC1** | | | | **PMAC2** | | | | **PMAC3** | | | | **PMAC4** | | | | **PMAC5** | | | |
| --- | --- | --- | --- | --- | --- | --- | --- | --- | --- | --- | --- | --- | --- | --- | --- | --- | --- | --- | --- | --- |
|  | Cell Line | | Tumour | | Cell Line | | Tumour | | Cell Line | | Tumour | | Cell Line | | Tumour | | Cell Line | | Tumour | |
| **D5S818** | 11 |  | 11 |  | 10 | 12 | 10 | 12 | 11 |  | 11 |  | 11 | 12 | 11 | 12 | 12 | 12 | 12 | 12 |
| **D13S317** | 8 |  | 8 |  | 13 |  | 7 | 12 | 11 |  | 11 |  | 8 | 12 | 8 | 12 | 13 | 13 | 8 | 13 |
| **D7S820** | 10 | 12 | 10 | 12 | 9 | 12 | 9 | 12 | 8 | 9 | 8 | 9 | 10 |  | 10 |  | 9 | 13 | 9 | 13 |
| **D16S539** | 9 | 13 | 9 | 13 | 11 | 12 | 11 | 12 | 11 | 12 | 11 | 12 | 12 |  | 12 |  | 11 | 12 | 11 | 12 |
| **vWA** | 14 | 17 | 14 | 17 | 17 | 18 | 17 | 18 | 15 | 17 | 15 | 17 | 17 | 19 | 17 | 19 | 16 | 17 | 16 | 17 |
| **TH01** | 7 |  | 7 |  | 6 | 9.3 | 6 | 9.3 | 9 | 9.3 | 9 | 9.3 | 6 |  | 6 |  | 6 | 9.3 | 6 | 9.3 |
| **TPOX** | 8 | 11 | 8 | 11 | 8 | 11 | 8 | 11 | 8 |  | 8 |  | 8 | 9 | 8 | 9 | 8 | 11 | 8 | 11 |
| **CSF1PO** | 11 | 12 | 11 | 12 | 10 | 11 | 10 | 11 | 10 | 12 | 10 | 12 | 11 |  | 11 |  | 11 | 11 | 11 | 11 |
| **Amelogenin** | X |  | X | Y | X |  | X |  | X |  | X |  | X | Y | X | Y | X | X | X | X |
| **D21S11** | 30.2 | 32 | 30.2 | 32 | 28 | 29 | 28 | 29 | 29 | 30 | 29 | 30 | 30 | 32.2 | 30 | 32.2 | 30 | 31.2 | 30 | 31.2 |
| **Match** | 94% | | | | 89% | | | | 100% | | | | 100% | | | | 95% | | | |

**Supplementary Table 3 Cell Line Culture Characteristics**

| **Cell Culture Characteristics** |  |  | **Line** |  |  |
| --- | --- | --- | --- | --- | --- |
|  | PMAC1 | PMAC2 | PMAC3 | PMAC4 | PMAC5 |
| Doubling Time (hrs) | 47 | 41 | 33 | 25 | 24 |
| Seeding Efficiency (%) | 1 | 6 | 9 | 33 | 24 |
| Migration (1 / Slope) | 155.6 | 140.4 | 73.9 | 61.8 | 48.7 |

**Supplementary Table 4 Parent tumour TMB, PD-L1 expression and CD3 TIL count**

|  | **PMAC1** | **PMAC2** | **PMAC3** | **PMAC4** | **PMAC5** |
| --- | --- | --- | --- | --- | --- |
| **TMB** (/Sample) | 457 | 108 | 52 | 106 | 86 |
| **PD-L1** (TPS) | < 1% | > 50% | < 1% | < 1% | 1 - 49% |
| **CD3 TILs** (/0.25mm^2^) | 381 | 263 | 360 | 43 | 281 |

*TMB – Tumour Mutational Burden; TILs – Tumour Infiltrating Lymphocytes; TPS – Tumour Proportion Score*

**Supplementary Table 5 Human ImmunoHisto- / Cyto- Chemistry Antibodies**

| **Antibody** | **Company / Cat #** | **Antigen Retrieval (Histo slides)** | **Secondary Antibody** |
| --- | --- | --- | --- |
| **p63** | Abcam, #ab97865 | Dako High pH Buffer | ImPRESS Rabbit |
| **CK5** | BioLegend, PRB-160P | 10mM Na Citrate Buffer, pH6 | ImPRESS Rabbit |
| **p16** | Roche, CINtec #9517 | 10mM Na Citrate Buffer, pH6 | ImPRESS Mouse |
| **Ki67** | Abcam, #ab16667 | 10mM Na Citrate Buffer, pH6 | ImPRESS Rabbit |
| **Anti-Human Mito** | Millipore, #MAB1273 | Dako High pH Buffer | ImPRESS Mouse |
| **p53** | Leica, NCL-p53-D07 | 10mM Na Citrate Buffer, pH6 | ImPRESS Mouse |
| **PDL1** | Ventana (SP263) | 1mM EDTA Buffer, pH 8.0 | ImPRESS Rabbit |
| **MHC-1** | Abcam, #ab70328 | 1mM EDTA Buffer, pH 8.0 | ImPRESS Mouse |

**Supplementary Table 6 PCR Oligonucleotides**

| **Type** | **Sequence** |
| --- | --- |
| Mycoplasma Forward | 5’-YGCCTGVGTAGTAYRYWCGC-3’ |
| Mycoplasma Reverse | 5’-GCGGTGTGTACAARMCCCGA-3’ |
| Cytochrome B Forward | 5’-AAAAAGCTTCCATCCAACATCTCAGCATGA-3’ |
| Cytochrome B Reverse | 5’-AAACTGCAGCCCCTCAGAATGATATTTGTC-3’ |
| HPV E6/7 Forward | 5'-TTGCAGATCATCAAGAACACGTAGA-3’ |
| HPV E6/7 Reverse | 5’ CAGTAGAGATCAGTTGTCTCTGGTTGC-3’ |

*Y = C or T; V = A or C or G; R = A or G; W = A or T; M = A or C*

**Supplementary Table 7 Seeding Density for Radiotherapy Clonogenic Assay**

| **RTx Dose**  **(Gy)** | **Seeding Density (x 10^3^)** | | | | |
| --- | --- | --- | --- | --- | --- |
|  | PMAC1 | PMAC2 | PMAC3 | PMAC4 | PMAC5 |
| 0 | 4 | 30 | 2 | 0.5 | 0.5 |
| 2 | 4 | 60 | 2 | 0.5 | 0.5 |
| 4 | 8 | 60 | 4 | 1 | 1 |
| 6 | 16 | 120 | 8 | 8 | 2 |
| 8 | 32 | 240 | 16 | 16 | 4 |
| 10 | 32 | 240 | 16 | 32 | 4 |

*Gy - Gray*
